# Enhancement of Immunosuppressive Activity of Mesenchymal Stromal Cells by Platelet-Derived Factors is Accompanied by Apoptotic Priming

**DOI:** 10.1007/s12015-022-10471-4

**Published:** 2022-11-22

**Authors:** Drenka Trivanovic, Noah Volkmann, Magdalena Stoeckl, Tobias Tertel, Maximilian Rudert, Bernd Giebel, Marietta Herrmann

**Affiliations:** 1 IZKF Research Group Tissue Regeneration in Musculoskeletal Diseases, University Hospital Würzburg, Würzburg, Germany; 2 Bernhard-Heine-Center for Locomotion Research, University of Würzburg, Würzburg, Germany; 3 Institute for Transfusion Medicine, University Hospital Essen, University of Duisburg-Essen, Essen, Germany; 4 Orthopaedic Department, University of Würzburg, Würzburg, Germany

**Keywords:** Hematoma, Platelet-rich plasma, Fibrin, Mesenchymal stromal cells, Immunomodulation, Apoptosis, Autophagy, Senescence, Extracellular vesicles, Metabolism

## Abstract

The pro-inflammatory phase of bone healing, initiated by platelet activation and eventually hematoma formation, impacts bone marrow mesenchymal stromal cells (MSCs) in unknown ways. Here, we created platelet-rich plasma (PRP) hydrogels to study how platelet-derived factors modulate functional properties of encapsulated MSCs in comparison to a non-inflammatory fibrin (FBR) hydrogel environment. MSCs were isolated from human bone marrow, while PRP was collected from pooled apheresis thrombocyte concentrates and used for hydrogel preparation. After their encapsulation in hydrogels for 72 h, retrieved MSCs were analyzed for immunomodulatory activities, apoptosis, stem cell properties, senescence, CD9^+^, CD63^+^ and CD81^+^ extracellular vesicle (EV) release, and metabolism-related changes. PRP-hydrogels stimulated immunosuppressive functions of MSCs, along with their upregulated susceptibility to cell death in communication with PBMCs and augmented caspase 3/7 activity. We found impaired clonal growth and cell cycle progression, and more pronounced β-galactosidase activity as well as accumulation of LC3-II-positive vacuoles in PRP-MSCs. Stimuli derived from PRP-hydrogels upregulated AKT and reduced mTOR phosphorylation in MSCs, which suggests an initiation of survival-related processes. Our results showed that PRP-hydrogels might represent a metabolically stressful environment, inducing acidification of MSCs, reducing polarization of the mitochondrial membrane and increasing lipid accumulation. These features were not detected in FBR-MSCs, which showed reduced CD63^+^ and CD81^+^ EV production and maintained clonogenicity. Our data revealed that PRP-derived hematoma components cause metabolic adaptation of MSCs followed by increased immune regulatory functions. For the first time, we showed that PRP stimuli represent a survival challenge and “apoptotic priming” that are detrimental for stem cell-like growth of MSCs and important for their therapeutic consideration.

**Graphical Abstract:**

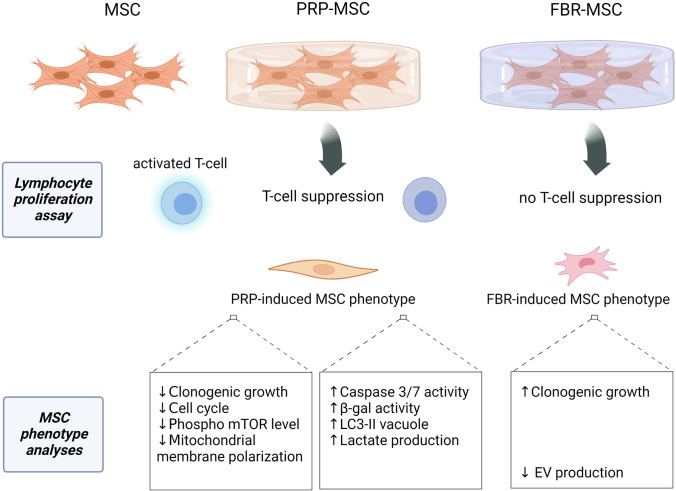

**Supplementary Information:**

The online version contains supplementary material available at 10.1007/s12015-022-10471-4.

## Background

As a complex sequence of events, bone healing includes three main stages: i) an initial inflammatory phase to remove damaged tissue, ii) a repair phase orchestrated by endothelial and bone marrow mesenchymal stromal cells (MSCs), and iii) a remodeling phase to form scar-free bone tissue. During the early pro-inflammatory phase, platelet degranulation and blood clot (hematoma) formation appear to be crucial for the recruitment of cells with regenerative properties and to initiate the healing processes at the injured site [1–3].

Beside prevention of blood loss, the hematoma formed during coagulation controls inflammatory and cellular processes, presenting a natural scaffold-like structure for tissue repair and regeneration. Using real-time in vivo fluorescence microscopy, it has been shown in mice that by means of the secretion of stromal-derived factor (SDF)-1α activated platelets recruit mouse bone marrow CD34^+^ and c-kit^+^Sca-1^+^Lin^−^ progenitors to sites of vascular injury, thereby supporting further the primary adhesion and migration of progenitor cells [4]. Moreover, endogenous platelet derived growth factors (PDGFs) can influence the proliferation capacity of MSCs ex vivo, as observed when the clonogenic growth of MSCs derived from the iliac crest of fracture patients was analyzed [5].

Based on these regenerative functions of platelets, platelet-derived products such as platelet rich plasma (PRP) have gained great interest in regenerative medicine. According to the functions of endogenous platelets and hematoma tissue, PRP is thought to entrap and support the proliferation and differentiation of surrounding endothelial cells and MSCs resulting in accelerated wound healing [6].

Blood- [7] or MSC-based [8] scaffolds/hydrogels have become attractive tools for investigating physiological or delayed bone healing processes as well as developing functional bone substitutes. This is particularly important since bone substitutes often require additional biological supplementation to support bone repair. Being packed with α and δ granules and lysosomes that contain coagulation and growth factors, antibacterial molecules and proteins with osteoinductive capacity, platelets and their concentrates (e.g. PRP) appear to be a meaningful option for the stimulation of fracture healing [9]. PRP-hydrogels have been used as an autologous hydrogel containing bioactive molecules with the potential to locally enhance tissue healing. Combining platelet-derived factors with MSCs can improve their immunoregulatory and osteogenic and chondrogenic ability [10]. Applied simultaneously in conjunction with MSCs, PRP-hydrogels were shown to exert beneficial effects in bone tissue regeneration in animal [11] and human models [12, 13]. However, results regarding the mechanisms of how PRP contributes to MSC activity and bone regeneration are still inconsistent. Platelet-derived factors interact with cells involved in inflammation, and may impact cell migration, differentiation, and extracellular matrix metabolism. Additionally, some PRP and platelet lysate (PL) elements, including fibrinogen, fibronectin and hyaluronan, can induce sterile inflammation by acting as damage-associated molecular patterns (DAMPs) and stimulate Toll-like receptors (TLR)4 and TLR2 [14].

Another attractive application of PRP and related products is the use as cell culture supplement in order to eliminate xenogenic products [15], but to date it is not completely clear how PRP/PL-based supplementation affects stemness, engraftment, immunomodulatory and secretory abilities of MSCs [16, 17]. Our previous study showed that conditioned medium (CM) from PRP-hydrogels contained a set of factors with pro-angiogenic, anti-inflammatory, pro-inflammatory and proliferation stimulatory activities. Results revealed an upregulation of genes involved in immunomodulation in MSCs stimulated with PRP‐ and bone fragment‐derived CM that likewise supported the immunosuppressive activity of CD146^+^ MSCs [18]. Reports by others indicated a successful culture of cells incorporated into PRP-gels, suggesting that the gel environment enhances viability and proliferation of encapsulated rat [19] or human bone marrow cells [20]. Thus, we anticipate that PRP-hydrogels might represent a reliable hematoma-mimicking model to investigate MSC activity and particularly their yet not explored survival and immunomodulatory potential. For this, we cultured MSCs in 3D PRP (PRP-MSCs) and fibrin (FBR)-hydrogels (FBR-MSCs) and followed their behavior after recovery. PRP-hydrogels induced a senescence-associated phenotype in MSCs, which was associated with increased caspase 3/7 activity, and a more effective immunosuppression activity of PRP-MSCs in vitro, when compared to FBR-MSCs. We found that this immunosuppressive effect of PRP-MSCs was associated with mTOR inhibition and a higher susceptibility to apoptotic cell death during co-culture with PBMCs. Although FBR-hydrogels did not display benefits in terms of immunoregulatory effects of MSCs, basic stem cell features (clonogenicity and differentiation) and cell homeostasis were better retained in FBR-MSCs compared to PRP-MSCs.

## Methods

### Human Subjects

Bone marrow (BM) samples were collected at the Department of Orthopedic Surgery, Wuerzburg (Ethical Committee approval 187/18) and the study was performed according to the Declaration of Helsinki and all participants signed informed consent. BM reaming samples from *acetabulum* were obtained as waste material from patients undergoing total hip arthroplasty surgery (19 patients, men = 10, women = 9, age 60.2 ± 14.6 years). Clinical evidences of osteoporosis, cancer or infectious disease were considered as exclusion criteria.

### Bone Marrow Cell Isolation and Expansion

Upon receiving, BM tissue was weighed and washed for several times with phosphate-buffered saline (PBS, Sigma-Aldrich, USA). The BM suspension was drained through 70 µm pore size strainers (Greiner Bio-One, Germany). BM low-density mononuclear cells were isolated using a Ficoll density gradient (1.077 ± 0.001 g/cm^3^, Sigma Aldrich, USA). After determination of cell numbers by counting in Trypan blue or Tuerk solution (both purchased from Sigma Aldrich), cells were cultured at 37˚C, 5% CO_2_ in a humidified atmosphere (standard conditions). Cells were seeded in medium (DMEM GlutaMax Ham’s F-12, D-glucose 3.15 g/L, Gibco, USA) supplemented with 1% of Penicillin/Streptomycin (Gibco, USA), 1% of Hepes (Sigma Aldrich, USA) and 10% of Fetal Calf Serum (FCS, BioCell, Germany) (standard growth medium, SGM) and 5 ng/mL recombinant human (rh) FGF (Promocell, Germany) at a density of 60,000 cells/cm^2^. After observed colony forming unit-fibroblastic (CFU-F) formation at day (10–14), clonogenic bone marrow mesenchymal stromal cells (MSCs) (passage 0, P0) were sub-cultured by re-seeding cells at a density of 3300 cells/cm^2^ into new cell culture dishes (Techno Plastic Products AG, TPP, Switzerland) up to passage P4 in SGM in standard conditions. Within P1 and P4, MSCs were passaged upon reaching 80–90% confluency. With respect to immunophenotype, clonogenic activity and differentiation, expanded cells were analysed according to the minimal criteria of the International Society of Cell and Gene Therapy (ISCT) [21]. For specific treatments, Staurosporin Biochemica (STS) (Applichem, USA) was added at concentration of 1 µM for 3 h, while chloroquine diphosphate (CQ) (Sigma Aldrich, France) was added at concentration of 50 µM for 12 h.

### Cultivation of MSCs in Platelet-Rich Plasma or Fibrinogen-Based Hydrogels

Platelet-rich plasma (PRP) was collected from pooled apheresis thrombocyte concentrates with tenfold increased platelet content (purchased from the Blood Donation Service of the Bavarian Red Cross, Wiesentheid, Germany). Thrombocyte concentrates were centrifuged at 2000 × *g* for 20 min at room temperature (RT). The upper supernatant, platelet poor plasma (PPP), was collected and used for specified PRP dilutions. The platelet-enriched fraction was re-suspended in the remaining volume of plasma and following steps were performed as described by Jalwiec et al. [20]. PRP with a final platelet concentration of 10^7^ platelets/µL, referred to as PRP100 in the following, and PPP were stored at -20˚C until use. To assess dose-dependent effects, we also included PRP50 in the study, containing 50% PRP and 50% PPP. MSCs were seeded in thrombin activated-hydrogels based on PRP or human plasma-derived fibrinogen (Sigma Aldrich, USA). A fibrinogen stock solution was prepared with a concentration 16.6 mg/mL and stored at -20˚C. Hydrogels were prepared by mixing PRP or human fibrinogen with human thrombin (Sigma Aldrich, USA) as the key proteins involved in blood clotting. Fibrinogen was reconstituted in aprotinin (Sigma Aldrich) at a concentration of 3000 KIU/ml, filtered through a 0.2 µm pore-size filter and diluted in sterile 0.9% NaCl to reach a final concentration of 5 mg/mL. Thrombin was dissolved in 40 mM CaCl_2_ and 5 μM ε-aminocaproic acid (Sigma Aldrich, Germany) added. MSCs (1000 cells/µL) were mixed with PRP or fibrinogen and loaded onto a thrombin drop (final concentration 5 U/mL) to induce polymerization and formation of PRP or pure fibrin (FBR) hydrogels. FBR-MSCs were cultured for 72 h, or less for specified conditions in SGM, while for PRP-MSCs, SGM without FCS was used.

### MSC Recovery and Cell Number Determination

In all experiments, MSCs were recovered from hydrogels or detached from tissue-culture plastic surface by collagenase NB4 (Nordmark, Germany) at a concentration of 1.25 U/mL by a 15–20 min incubation at 37˚C in a humidified atmosphere. The enzymatic reaction was stopped by adding SGM and cells were washed out by two centrifugation steps at 600 g for 5 min at RT. Subsequently, cell suspensions were filtered through 70 µm pore size strainers to be purified from gel pieces. Afterwards, cells were re-suspended in SGM, counted and used for the experiments. The number of recovered cells after their cultivation in hydrogels or upon specific treatments was determined by the Trypan-blue exclusion test. For assays where cells were additionally cultured after retrieval, MSCs were re-seeded at a density of 7 × 10^4^ cells/well and cell counting was performed after 24, 72 and 168 h.

### Colony Forming Efficiency

MSCs retrieved from hydrogels or upon the specified treatments, were seeded at a density of 250 cells/well in a 6-well plate (TPP) in SGM with or without rhFGF and cultured for 14 days in standard conditions. CFU-F efficiency was determined by scoring the total number of colonies containing more than 50 cells after staining with 0.5% Crystal Violet in methanol (Sigma Aldrich, USA).

### Isolation and Storage of Peripheral Blood Cells

Peripheral blood mononuclear cells (PBMCs) were isolated from buffy coats of healthy donors purchased from the local transfusion center (Blood Donation Service of the Bavarian Red Cross, Wiesentheid, Germany) using Ficoll density gradient (1.077 ± 0.001 g/cm^3^) centrifugation at 800 × *g* for 30 min at RT. For this study, randomly selected PBMCs from in total 10 individual donors were used. After gradient separation, mononuclear cells were washed in PBS, and erythrocytes lysed by using erythrocyte lysis buffer (Thermo Fisher, USA). PBMCs were frozen at a concentration of 10^7^ cells/mL in freezing medium containing 90% FCS and 10% of DMSO (Sigma Aldrich, USA) in liquid nitrogen. For thawing, RPMI GlutaMax (Gibco, USA) supplemented with 1% of Penicillin/Streptomycin, 1 mM Hepes buffer, and 10% of FCS (Lymphocyte growth medium, LGM) was used. PBMC stored for up to 1 month were used for experiments.

### Co-culture of MSCs with PBMCs: Proliferation, Cytokine Production and Apoptosis Assays

#### Proliferation and Phenotype of T-lymphocytes

PBMCs were labeled with 5 µM of 5(6)-Carboxyfluorescein-diacetate *N*-succinimidyl-ester (CFSE, λ_excitation_ 492 nm; λ_emission_ 517 nm, Sigma-Aldrich, USA) for 10 min in PBS w/o FCS in the dark at room temperature. The staining was stopped by adding the fivefold volume of PBS with FCS. PBMCs were seeded in 24-well plates at a density of 1 × 10^6^ cells/well. For PBMC activation, phytohemagglutinin-L (PHA-L) (Thermo Fisher Scientific, USA) was added at final concentration of 2 µL/mL in LGM. For co-cultures, MSCs were added at ratio 1:10 in LGM and cells were additionally incubated for the next 72 h before cells were collected and stained for flow cytometry analyses.

#### Interferon (IFN)-γ Production

PBMCs were stimulated with phorbol 12-myristate 13-acetate (PMA, 10 ng/mL) and ionomycine (1 µg/mL) for 24 h (both from Sigma Aldrich, USA) in LGM. Where indicated, MSCs were added at ratio 1:10. After overnight culture in standard conditions, secretion inhibitor brefeldin A (10 µg/ml, Sigma Aldrich, USA) was added to the cells. After 4 h, cells were collected and stained for flow cytometry analyses.

#### Apoptosis Assay

PBMCs were seeded in 12-well plates at a concentration of 2 × 10^6^ cells/well in 2 mL of LGM with the corresponding amount of PHA-L. We increased the amount of cells in this assay to have the possibility to more accurately explore MSCs in co-culture. Upon cultivation in hydrogels or standard 2D conditions, MSCs were labeled with cell permeable CellTracker™ Green CMFDA fluorescent dye (λ_excitation_ 492 nm; λ_emission_ 517 nm, Thermo Fisher, USA). For this, cells were incubated in pre-warmed CellTracker working solution at a concentration of 5 µM in standard medium without FCS and incubated for 15 min at 37°C in the dark. Staining was stopped by adding standard medium with FCS and cells were washed two times by centrifugation at 800 × *g* for 5 min. The number of cells was determined by Trypan blue exclusion staining. CellTracker + MSCs at a concentration of 2 × 10^5^ cells/well were added to PHA-stimulated PBMCs seeded into 12-well plates. After 3 days, cells were collected and stained for flow cytometry as described below.

### Flow Cytometry: Cell Phenotype, Apoptosis and Cell Cycle Analyses

Cells were immunophenotyped combining a panel of lymphocytic and stromal cell markers. A list of fluorescent conjugated anti-human antibodies used for cytometry including clone, conjugate, and catalogue number is provided in STable 1. Washing steps were performed using PBS without ions and supplemented with 5% of FCS (flow cytometry buffer) and centrifugation at 600 × *g* for 5 min. Fixable viability dye staining was followed by incubation with antibodies against surface antigens (added at concentrations recommended by the manufacturer) at 4°C for 45 min. Staining of intracellular antigens was performed upon fixation and permeabilization of cells using Foxp3/Transcription Factor Staining Buffer Set according to the manufacturer’s protocol (Invitrogen, eBioscience, USA). For the investigation of apoptosis, after viability staining and incubation with surface marker antibodies, cells were washed in buffer (PBS containing Ca^2+^ and Mg^2+^ with 1% FCS) and subsequently in 1 × Annexin V-binding buffer (Invitrogen, USA). Cells were re-suspended in 1 × Annexin V binding buffer at a concentration of 1 to 5 × 10^6^ cells/mL and 5 µL of PE-conjugated Annexin V was added to the final 100 µL cell suspension. Cells were incubated 10–15 min at room temperature, protected from light. After washing with 1 × Annexin V-binding buffer, cells were resuspended in buffer and analyzed by flow cytometry. For cell cycle analyses, after surface marker staining, cells were stained with Hoechst 33342 (Sigma Aldrich, USA) at a concentration of 5 ng/mL in flow cytometry buffer for 20 min at 4°C. Afterwards, cells were fixed with 4% PFA for 6 min, rinsed and re-suspended in buffer for further analyses. To analyze the change in mTOR phosphorylation (Ser2448), MSCs were seeded in hydrogels or exposed to specified treatments and cultured for 24 or 72 h. Afterwards, cells were retrieved from gels and processed as stated above. Fixation and permeabilization of cells were performed using Foxp3/Transcription factor staining buffer kit according to the manufacturer’s protocol (Invitrogen, eBioscience, USA). Cells were stained with PE-Cy7-conjugated anti-phospho-mTOR monoclonal (anti-human/mouse) antibody (eBioscience) to analyze the mTOR phosphorylation level. Unstained samples were used as negative control. Multicolor flow cytometry was performed using a BD LSR II (BD Bioscience, USA) or Attune Nxt Flow Cytometer (Thermo Fisher). Compensation was performed with single-stained compensation beads (BD Bioscience, USA) in PBS using the same antibody dilutions as for the cell staining. All data analyses were performed using FlowJo V10 software (TreeStar, USA).

### Imaging Flow Cytometry for EV Screening

After recovery from hydrogels or specified treatments, MSCs were seeded at a density of 1 × 10^5^ cells/well into 24-well plates in 1 mL of complete LGM (containing FCS), to replicate the same conditions that MSCs experienced in co-culture experiments. Cells were cultured for 72 h, and thereafter MSC-free conditioned media (CM) of the cultured cells was collected. For cryopreservation of the CM, cells and larger debris were removed from the CM by 2,000 × *g* centrifugation of CM for 15 min. Subsequently, CM was stored at -80°C until usage. After thawing, EV contents were analyzed in thawed CM by imaging flow cytometry exactly as described previously [22, 23]. Briefly, after thawing, CM were filtered through 0.22 µm filters (Sartorius, Göttingen, Germany). CM aliquots of 20 µL or 40 µL were transferred into individual wells of 96-well U-bottom plates (Corning Falcon, USA) and stained with anti-human CD9 PE (EXBIO, Czech Republic) or, anti-human CD63 APC (EXBIO) or and anti-human CD81 FITC (Beckman Coulter, USA) antibodies for 1 h, respectively. Complete and FCS-depleted LGM medium served as background control. Before analysis, sample volumes were adjusted with PBS to final volumes of 200 µL. Samples were analyzed for 5 min on an AMNIS ImageStreamX Mark II Flow Cytometer (AMNIS/Luminex, Seattle, WA, USA). All data were acquired at 60 × magnification at low flow rate (0.3795 ± 0.0003 μL/min, determined directly from the system) and with the removed bead option disabled exactly as described previously [22, 23].

### Immunofluorescence Microscopy

For immunofluorescence analyses, MSCs were recovered from hydrogels and seeded on glass coverslips to adhere overnight. MSCs were fixed in 4% paraformaldehyde or 75% methanol (for anti-LC3B antibody staining) according to the antibody manufacturer’s recommendation and stored in the dark at 4°C in 0.1% NaN_3_ (Sigma Aldrich, Israel) in PBS. Cells were permeabilized with 0.1% Triton-X100 (Sigma Aldrich, USA) in PBS for 10–15 min at room temperature and incubated with blocking solution: PBST (0.1% Tween 20 (Sigma Aldrich), 1% bovine serum albumin (BSA, Roche, Germany) in PBS (Sigma Aldrich)) for 30 min at 4°C. The cells were incubated with primary antibodies for lamin B1 (dilution 1:1000, R&D Systems, USA), LC3B (dilution 1:1000, Cell Signaling Technology, USA) and COX-2 (1:100, Santa Cruz Biotechnology, USA) in PBST overnight at 4°C. After three washing steps in PBS, cells were incubated with secondary antibodies diluted in PBST for 1 h at RT in the dark. Secondary antibodies were used as follows: anti-rabbit efluor488 (1:300 dilution, Abcam, USA) and anti-mouse efluor594 (1:300 dilution, Abcam, USA) and anti-mouse efluor488 (1:300 dilution, Abcam, USA). For the last 10 min, 4′,6-diamidino-2-phenylindole dihydrochloride (DAPI, Sigma Aldrich) or 10 ng/mL or Hoechst 33342 at 5 ng/mL (Sigma Aldrich) was added for DNA staining. Coverslips were mounted with a drop of Vectashield Antifade Mounting Medium (Vector Laboratories, USA) onto microscopy slides and stored in the dark at 4°C. For detection of acidic vesicular organelles, cells were incubated with 50 µM Acridine Orange (Sigma Aldrich, USA) solution in PBS for 10 min at RT in the dark. Acridine orange is a cell-permeable green fluorophore that can be protonated and trapped in acidic vesicular organelles [24], which can increase after autophagy induction, emissions at 530 nm (green) and 680 nm (red) were used for identification of acidified (red) vesicles. For neutral lipid accumulation, BODIPY™ 493/503 (4,4-Difluoro-1,3,5,7,8-pentamethyl-4-bora-3a,4a-diaza-*s*-indacene, Invitrogen, USA) was added at a final concentration of 10 ng/mL. For negative controls, cells stained for DNA or with secondary antibodies only were analyzed. Data regarding primary and secondary antibodies as well as probes are available in STable 1. Fluorescence was checked by fluorescene microscopy (DMi8, Leica Microsystems).

### Preparation of Hydrogel-Derived Conditioned Media and Western Blot Analyses

To evaluate the effects of soluble factors released from PRP or FBR hydrogels on MSCs, we prepared conditioned media (CM) collected after 24 h of incubation of hydrogels. PRP or FBR hydrogels were performed as described above. After polymerization, LGM without FCS was added and gels were incubated in standard conditions. CM were collected, filtered through a 0.2 µm pore-size filter and stored at -80°C. CM collected after 24 h was used at a concentration of 10% for MSC treatment. MSC were seeded in 6-well plates at a density of 10^5^ cells/well and cultured in SGM until 80% of confluency was reached. For the treatment, LGM without FCS, supplemented with 1% ITS (Sigma Aldrich) and 10% of CM was used. Cells were treated for 24 h and subsequently lysed using RIPA buffer (Thermo Fisher Scientific) supplemented with proteinase inhibitor cocktail and 5 mM EDTA (Thermo Fisher Scientific). The total amount of protein was quantified using the Pierce BCA kit (Thermo Fisher Scientific). Cell lysates were denatured and reduced using 5 × Laemmli Buffer with 2-mercaptoethanol at 95°C for 5 min. 10 or 30 (for mTOR) µg of total protein was loaded on 10% or 6% (for mTOR) acrylamide gels followed by sodium dodecyl sulfate polyacrylamide gel electrophoresis (SDS-PAGE). Proteins were transferred to the 0.22 µm pore size nitrocellulose membrane (GE Healthcare). Membranes were blocked with 5% BSA (Roche) or non-fat dry milk (Carl Roth) in Tris (Applichem)-buffered saline with 0.1% Tween-20 (Sigma-Aldrich). Membranes were incubated with primary antibodies: LC3B, β-actin, phospho- and total-mTOR, phospho- and total-AKT (all at a dilution of 1:1000) overnight at 4°C, followed by an incubation with anti-rabbit or anti-mouse immunoglobulin G (IgG) antibody conjugated with horseradish peroxidase (both diluted 1:1000). Data regarding primary and secondary antibodies are available in STable 1. Protein bands were visualized by using Chemiluminescent HRP substrate (Biozym) or WesternSure® PREMIUM Chemiluminescent Substrate (Licor) and images acquired using the FluorChem Q Imaging System (Cell Biosciences) or C-DiGit® Blot Scanner (Licor).

### Measurement of Glucose Consumption and Lactate Production

Media collected upon 72 h of cultivation of MSCs in hydrogels was filtered through a 0.22 µm pore size filter (Corning) and stored at -80˚C. Analyses of glucose and lactate levels were performed by using a Cedex Bio analyzer (Roche, Germany). Obtained values were subtracted for the values of LGM incubated alone or with hydrogels without cells, and subsequently normalized to the cell number determined by counting MSCs upon recovery from gels.

### Mitochondrial Membrane Potential and Biomass

The mitochondrial inner membrane potential and mitochondrial mass were measured in MSCs cultured in PRP or FBR-based gels for 72 h. To determine the mitochondrial inner membrane potential 3 × 10^5^ cells per condition were incubated with 100 nM MitoTracker CMXRos (Thermo Fisher Scientific, USA) in SGM without FCS for 30 min in the dark at 37°C. Cells were washed with PBS containing 5% of FCS and 100 mM Hepes. The cells were rinsed, and fluorescence detected by a BD LSR II (BD Bioscience) or Attune Nxt Flow Cytometer (Thermo Fisher). Subsequently, to each tube 10 μM of uncoupling and depolarizing agent, carbonyl cyanide 4-(trifluoromethoxy)-phenylhydrazone (FCCP, Sigma Aldrich, USA) was added and incubated for 10 min in the dark at 37°C. The samples were rinsed, and fluorescence intensity measured again. The mitochondrial inner membrane potential was obtained for each sample as absolute difference in detected fluorescence before and after addition of FCCP. The mitochondrial mass was detected by incubation of 5 × 10^4^ cells/glass coverslip. 3 replicates per condition (treatment group) were seeded. Cells were stained with MitoTracker Green fluorescent probe (MTG) (Cell Signaling Technology, USA) at a final concentration of 10 nM for 30 min in SGM without FCS in the dark at 37°C. The cells were rinsed with PBS with 5% FCS and 100 mM Hepes. Coverslips were mounted onto microscopic slides. Mitochondrial fluorescence was checked by fluorescent microscopy (DMi8, Leica Microsystems).

### Total ATP Content Measurements

After recovery from hydrogels or specified treatments, MSCs were seeded at a concentration of 3 × 10^3^ cells/well into opaque 384-well plates with clear bottom. After 24, 72 and 168 h, ATP content was measured by adding CellTiterGlo® reagent (Promega, USA). Freshly prepared ATP disodium salt (Sigma Aldrich, USA) standards were used to calculate the ATP content. Values were subtracted for background luminescence of SGM incubated without cells. Luminescence was determined by using the Tecan reader (Tecan Trading AG).

### Caspase 3/7 Assay

After recovery from hydrogels or specified treatments, MSCs were seeded at a concentration of 5 × 10^3^ cells/well into opaque 96-well plates with clear bottom. After 24 or 72 h, caspases 3/7 cleavage activity was measured by adding Caspase 3/7Glo® reagent containing thermostable luciferase (Promega, USA). After 3 h of incubation at RT, luminescence was determined by using a Tecan reader. Values were corrected for luminescence of SGM incubated without cells. STS was applied as apoptosis promoter at a concentration of 1 µM for 3 h.

### β-galactosidase Staining

Cultured MSCs were seeded at a concentration of 6 × 10^4^ cells/well in 24-well plates in SGM. After 24 h, activity of β-galactosidase was visualized by Senescence Cells Histochemical Staining Kit (Sigma-Aldrich, USA) according to the manufacturer’s protocol. Cells were observed by light microscopy (Leica Microsystems). The percentage of β-galactosidase positive cells was determined by scoring the number of β-gal positive cells in the total number of counted cells for at least 3 fields of view per replicate by using the 10 × objective.

### RNA Extraction and qRT-PCR

For RNA extraction, MSCs were seeded in 6-well plates and cultivated under corresponding conditions. Total mRNA was extracted and purified using a Nucleo Spin® RNA Kit (Machery-Nagel, Germany) according to the manufacturer’s instructions. The RNA concentration was determined using the NanoQuant plate of a Tecan Reader (Tecan Trading AG) and 1 µg of RNA was used for cDNA synthesis by Oligo(dT) primers, M-MVL Reverse transcriptase, dNTP mix and 5 × Reaction Buffer (all purchased from Promega, USA). Real-time qPCR was performed using BioRad CFX96 analyzer (BioRad, USA), containing GoTaq master mix ® (Promega, USA) in accordance with the manufacturer’s guidelines. Primer sequences (all from Biomers, Germany) are provided in STable 2. mRNA expression was analyzed by SYBR green-based qPCR in 20 μL duplicate reactions using 2 μL of cDNA template and primers at a concentration of 250 nM. Expression of all target genes was determined at a primer annealing temperature of 40 °C after 40 cycles and normalized to the expression of β2-microglobulin (*B2m*). Relative expression values were determined using the 2^−ΔΔct^ method.

### Statistical Analysis

All obtained data were analyzed for normal distribution within each experimental group using the D'Agostino & Pearson normality test where possible due to sample size. The number of investigated individual MSCs donors (biological replicates) is indicated as *n* for each experiment. The statistical significance of the differences in the means of experimental groups was determined by appropriate non-parametric models and a post hoc test for multiple comparisons, as indicated in the figure legends. Bars present means ± SD. A p-value < 0.05 was considered as statistically significant. All statistical analyses were performed using GraphPad Prism 9.2.0 software (USA) and details are described in the figure legends.

## Results

### PRP-Hydrogel Stimulates Immunosuppressive Functions of MSCs

**Fig. 1:**
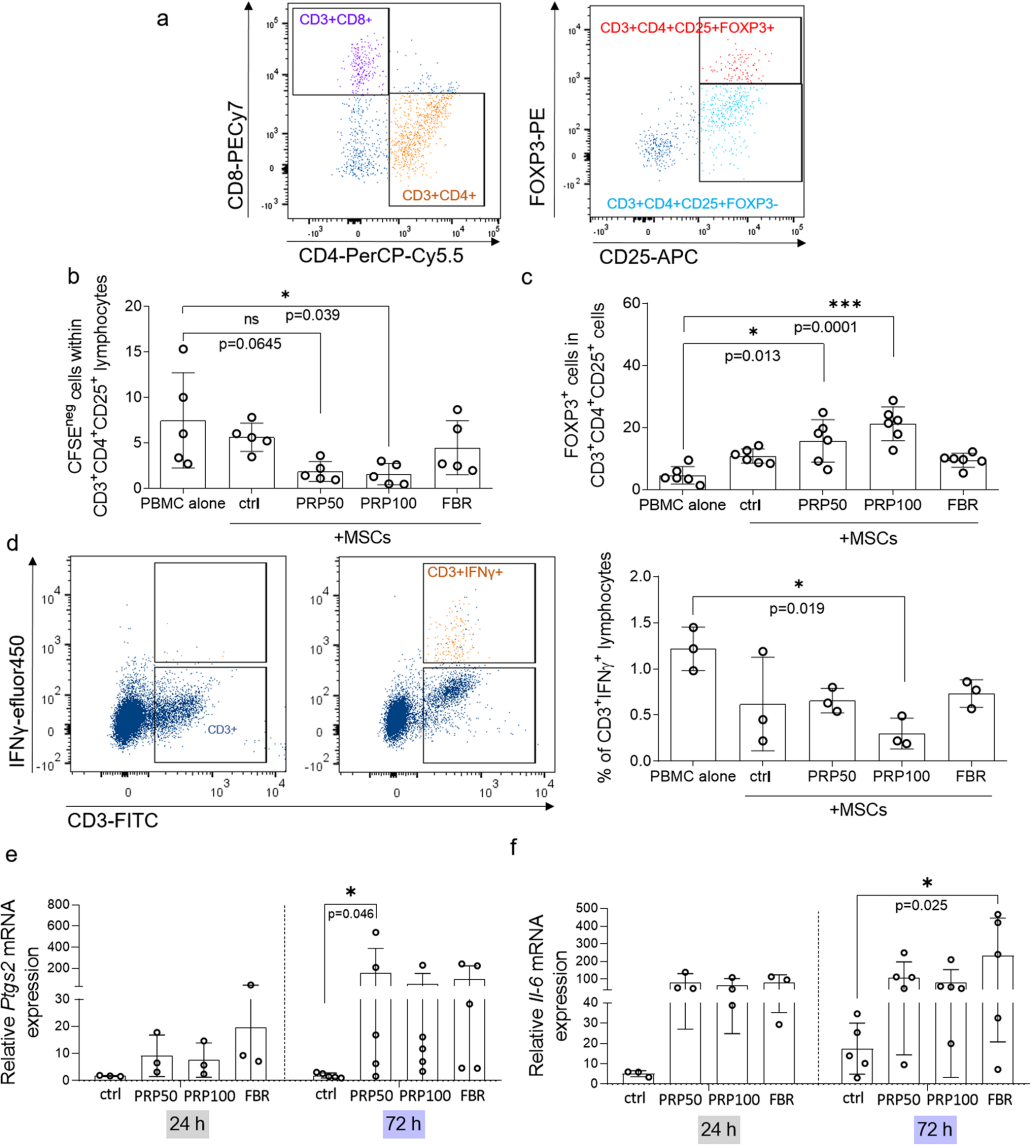
PRP-hydrogels increase immunosuppressive activity of MSCs. (**a**) Gating strategy of T lymphocytes within PBMCs. Percentage of: (**b**) CFSE^neg^ (n_MSC_ = 5, n_PBMC_ = 5) and (**c**) FOXP3^+^ cells (n_MSC_ = 6, n_PBMC_ = 4) within CD3^+^CD4^+^CD25^+^ lymphocytes determined after 72 h of PBMC culture alone or with MSCs. (**d**) Estimation of IFN-γ-producing cells within CD3^+^ PBMCs analyzed after 24 h of stimulation in absence or presence MSCs. Gating strategy (left part) and percentage (right part) (n_MSC_ = 3, n_PBMC_ = 3) are presented. PBMC donors were selected randomly and each MSC donor was tested with PBMCs obtained from at least 2 individual donors. Gene expression of (**e**) *Ptgs2* and (**f**) *Il-6* determined at 24 h (*n* = 3) and 72 h (*n* = 5) upon culture of MSCs in standard conditions or hydrogels. Relative values to MSC cultures in SGM without FCS were calculated for each group. Results are presented as mean ± SD. For comparison between groups, Kruskal–Wallis and two-way ANOVA with Bonferroni's multiple comparisons test was used to compare to control and within groups in respect to the unexposed control sample, where *p < 0.05 and ***p < 0.001 indicate significant differences

Our previous study suggested that conditioned media derived from bone fragments and PRP-hydrogels can stimulate immunoregulatory properties of MSCs [18]. Here, we investigated whether MSCs recovered from PRP-hydrogels, closely mimicking the hematoma environment, display a similar activity to modulate the phenotype and proliferation of T lymphocytes (Fig. 1a–d, SFig. 1a). Since co-cultures were performed in LGM, we tested viability of MSCs exposed to LGM with or w/o PHA for 72 h and did not observe any significant changes in MSC viability nor morphology (SFig. 1b–d). To investigate T lymphocyte proliferation, CFSE-stained PBMCs were stimulated with PHA and seeded in the presence or absence of MSCs. Non-activated and PHA-activated PBMCs cultured alone were used as control. 72 h past seeding, PBMCs were harvested and explored by flow cytometry for their CFSE intensity as indicator of proliferation. Cytometry analyses of alive CFSE^neg^ PHA-activated PBMCs, referring to actively proliferating cells, showed that PRP-MSCs (at 50% and 100% concentration) suppressed the proliferation of CD3^+^CD4^+^CD25^+^ PBMCs (Fig. 1a, b) from 7.47 ± 5.23% in PBMC cultured alone to 1.86 ± 1.09% (*p* = 0.0645) or 1.58 ± 1.17% (*p* = 0.039) in co-culture with PRP50-MSCs or PRP100-MSCs, respectively. In addition, PRP-cultured MSCs increased the proportion of FOXP3^+^ cells within the CD3^+^CD4^+^CD25^+^ population (Fig. 1a, c), from 4.60 ± 2.80% in alive PHA-stimulated PBMCs cultured alone to 15.68 ± 6.84% (*p* = 0.013) or 21.18 ± 5.46% (*p* = 0.0001) in co-culture with PRP50-MSCs or PRP100-MSCs, respectively. Comparable effects were not observed within the CD8^+^ lymphocyte compartments (data not shown). In addition, MSCs cultured in 100% of PRP-hydrogel, decreased the frequency of lymphocytes producing IFN-γ, CD3^+^IFN-γ^+^ cells, within PHA-stimulated PBMCs from 1.22 ± 0.23% to 0.3 ± 0.1% (Fig. 1d). In contrast, MSCs cultured in FBR-hydrogel did not show any significant impact on T cells. Interestingly, an increased expression of *Ptgs2* at gene (Fig. 1e), as well as slightly at protein level (SFig. 2a) in MSCs cultured in PRP-hydrogels was found, while only FBR-MSCs showed a statistically significant increased *Il-6* mRNA expression after 72 h when compared to the control group (Fig. 1f). These results indicate that the PRP environment stimulates immunoregulatory activities in MSCs, while FBR does not show this potential.

### PRP-Hydrogel Enhances MSCs’ Susceptibility to Cell Death in Direct Communication with PBMCs

**Fig. 2:**
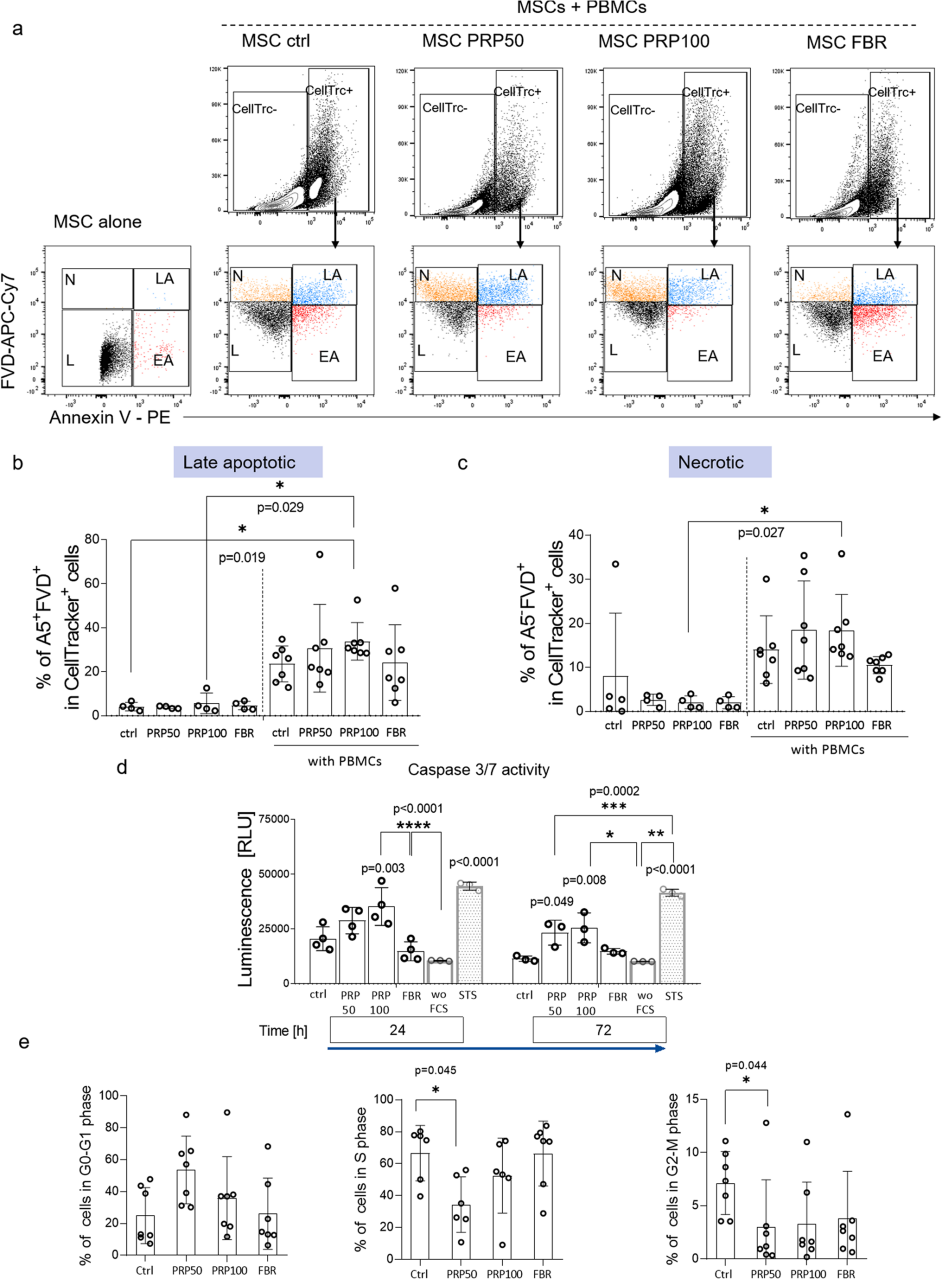
PRP-hydrogel supports apoptotic cell death in MSCs and inhibits cell cycle progression. (**a**) Gating strategy applied for analyses of apoptosis in MSCs co-cultured with PBMCs. The selection of four subpopulations (live, early apoptotic (EA), late apoptotic (LA) and necrotic (N)) is indicated. Percentage of (**b**) late apoptotic and (**c**) necrotic MSCs cultured alone or in presence of PHA-stimulated PBMCs. (**d**) Activity of caspases 3/7 evaluated 24 h and 72 h upon their recovery from hydrogels (*n* = 3–4). Results are presented as mean ± SD. **e**) Cell cycle phases in MSCs (*n* = 7) and changes in G0-G1, S and G2-M rates (*n* = 7). For comparison between groups, Kruskal–Wallis and two-way ANOVA with Bonferroni's multiple comparisons test was used to compare to the control and within groups in respect to the unexposed control sample, where *p < 0.05, **p < 0.01, ***p < 0.001 and ****p < 0.0001 indicate significant differences

Next, we examined the behavior of CellTracker Green-labeled MSCs during their co-culture with activated PBMCs. Our first results indicated that MSCs spontaneously undergo cell death when cultured with PHA-stimulated PBMCs, while this did not occur when MSCs were cultured alone (Fig. 2a–c). Results show that MSCs cultured in PRP-hydrogels were more prone to undergo apoptotic cell death. We observed increased frequencies of AnnexinV^+^FVD^+^ (late apoptotic) (Fig. 2a, b) and AnnexinV^−^FVD^+^ (necrotic) MSCs (Fig. 2a, c) with statistical significance when compared to MSCs from standard culture and/or PRP-MSCs cultured alone. In contrast, FBR-MSCs showed a similar susceptibility to cell death as the control group (MSCs cultured in LGM) (Fig. 2a–c). Apoptosis is the mode of cell death program [25] controlled by the members of the caspase family of cysteine proteases. Thus, we analyzed the production of executioner caspases 3 and 7 and found their significant upregulation in PRP-MSCs, which was comparable to the effect of incubation with the apoptosis inductor, STS (Fig. 2d). From these results we concluded that MSCs might be exposed to “apoptotic priming” during their 72-h maintenance in the PRP-hydrogel, resulting in an increased level of apoptosis of MSCs upon co-culture with PBMCs, while FBR-MSCs stayed indifferent in this context. In addition, we analyzed cell cycle progression immediately after the recovery of MSCs from hydrogels and results showed a significantly lower frequency of PRP50-MSCs in proliferating S and G2-M phase, indicating that PRP-MSCs survived in resting G0-G1 phase of the cell cycle (Fig. 2e). Interestingly, we did not observe significant changes in MSCs cultured in PRP100 or FBR hydrogels. To test the short-term proliferative potential, we followed the proliferation of MSCs over the 72-h culture in PRP- and FBR-hydrogels, and we did not observe any significant effect of the hydrogel environment on MSC proliferation after 1, 3 or 7 days after recovery (SFig. 2b) nor on the surface marker expression of MSCs (STable 3). Therefore, we concluded that “apoptotic priming” induced by the PRP-hydrogel environment, might lead to enhanced MSC death during their communication with PBMCs.

### β-galactosidase Activity and LC3-II-Positive Structures were more Pronounced in PRP-MSCs, while Extracellular Vesicle Production and p16 Expression was Reduced in FBR-MSCs

**Fig. 3:**
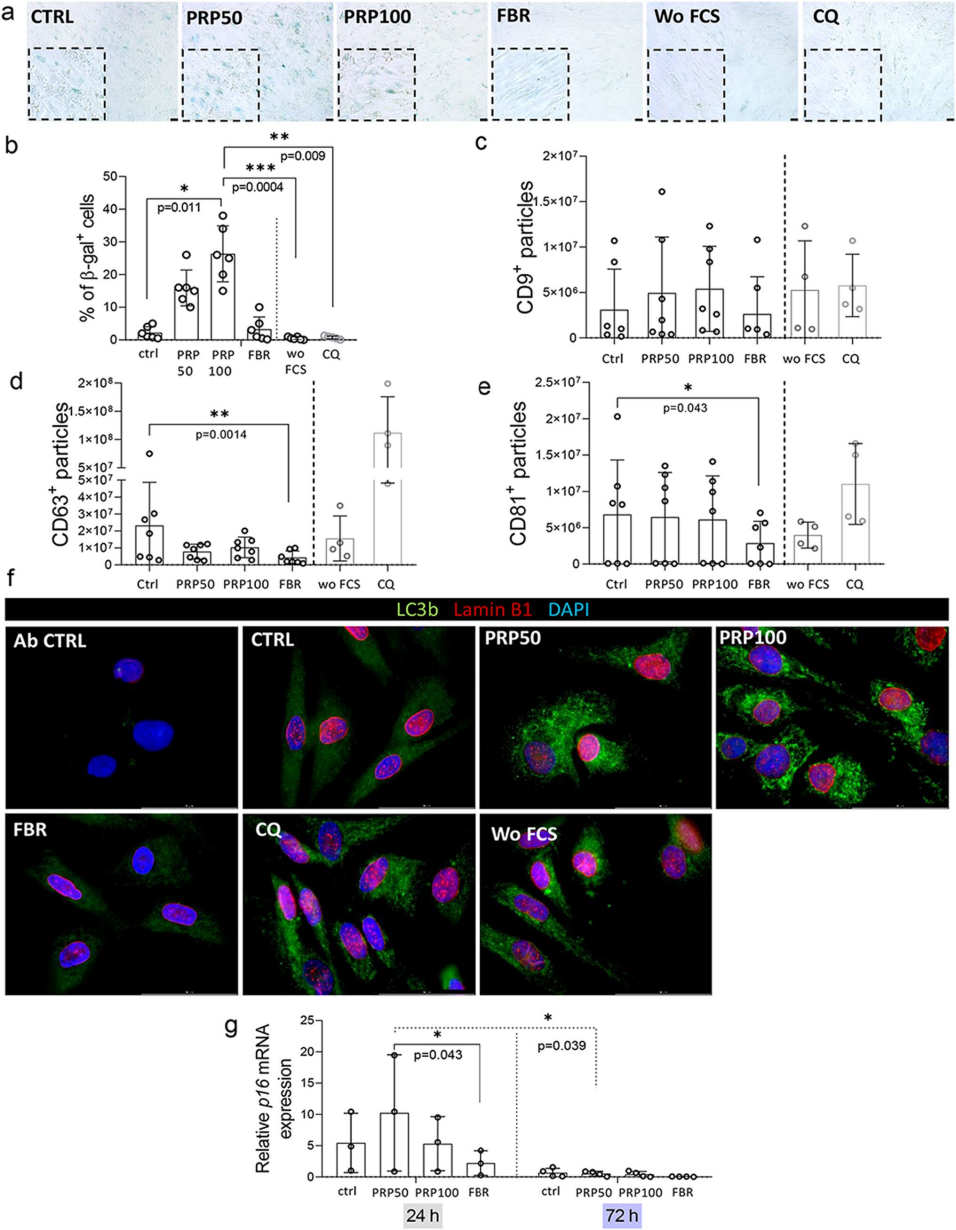
PRP-hydrogels contribute to a senescent profile and autophagosme accumulation in MSCs, while FBR reduce their extracellular vesicle production. (**a**) Representative photos of β-galactosidase staining are shown (*n* = 6). Scale bar = 50 µm. (**b**) Percentages of β-galactosidase + MSCs (*n* = 6). Percentage of (**c**) CD9^+^, (**d**) CD63^+^ and (**e**) CD81^+^ particles produced by MSCs 72 h upon their recovery from hydrogels or indicated conditions (*n* = 7). (**f**) Expression of autophagosomal protein LC3b by MSCs recovered from hydrogels. Representative images are shown (*n* = 4). Scale bars = 50 µm. (**g**) Gene expression of *p16* determined at 24 h (*n* = 3) and 72 h (*n* = 4) upon culture of MSCs in standard conditions or hydrogels. Relative values to MSC culture in SGM without FCS were calculated for each group. Results are presented as mean ± SD. For comparison between groups, Kruskal–Wallis and two-way ANOVA with Bonferroni's multiple comparisons test was used to compare to the control and within groups in respect to the unexposed control sample, where *p < 0.05, **p < 0.01 and ***p < 0.001 indicate significant differences

Survival of cells in stressful environments is associated with cellular damage that can initiate cell death program or prompt the cells to become senescent and persist until their removal by the immune system [26]. After the observed changes in cell cycle progression, we investigated β-galactosidase activity in MSCs as one of the hallmarks of senescent cells. Results showed that MSCs cultured in PRP-hydrogels displayed a higher percentage of β-gal^+^ cells, when compare to the other tested groups (Fig. 3a, b). This effect was not observed for FBR-MSCs, nor for MSCs cultured in absence of FCS. In these groups β-gal activity was comparable to that observed upon chloroquine hydrochloride (CQ) treatment, a compound with senolytic activity [27] (Fig. 3a, b), but also a promoter of extracellular vesicle production [28]. EVs have been suggested to mediate immunosuppressive [29] and bone regenerative activities of MSCs [30]. Thus, we investigated whether EV production is altered by hematoma mimicking hydrogels. We determined frequencies of particles positive for EV-related markers (SFig. 2c) without pre-enrichment and by using imaging flow cytometry. The screening showed that the accumulation of CD63^+^ and CD81^+^ particles (objects) was lowest in the FBR-MSC group (Fig. 3d, e). We did not observe changes in the presence of CD9^+^ particles within the experimental groups (Fig. 3c). On the other side, although without statistical significance, the frequencies of CD63^+^ and CD81^+^ particles were highest when MSCs were pretreated with CQ as “positive control” for EV production (Fig. 3d, e). Collectively, our results demonstrated that hematoma mimicking environments did not alter EV production, while surprisingly, FBR-hydrogels prevented EV secretion. We assumed that these effects might be related to the regulation of cell homeostasis and autophagosome degradation. To address this, we investigated changes in lamin B1 expression and a lipidated form of the microtubule-associated protein light chain 3B (LC3B), where LC3-II positive structures are also referred to as autophagosomes (Fig. 3f). Obtained results indicated that the PRP-induced senescent profile of MSCs was associated with prevented autophagosome degradation, but without changes in nuclear envelope organization defined by lamin B1 expression. Effects of PRP were similar to those induced in FCS-free conditions and treatment with CQ. From analyses of *p16* mRNA expression (Fig. 3g), it can be concluded that the PRP-hydrogel environment upregulated its’ expression, when compared to FBR-hydrogel. This might be related to the higher β-gal^+^ cell percentage and the impaired cell cycle progression in PRP-MSCs. On the other side, FBR-hydrogel did not elevate *p16* mRNA expression and reduced EV production by MSCs, without appearance of β-gal^+^ cells or LC3-II positive structures.

### PRP-Derived Stimuli Regulate AKT and mTOR Pathways in MSCs

**Fig. 4:**
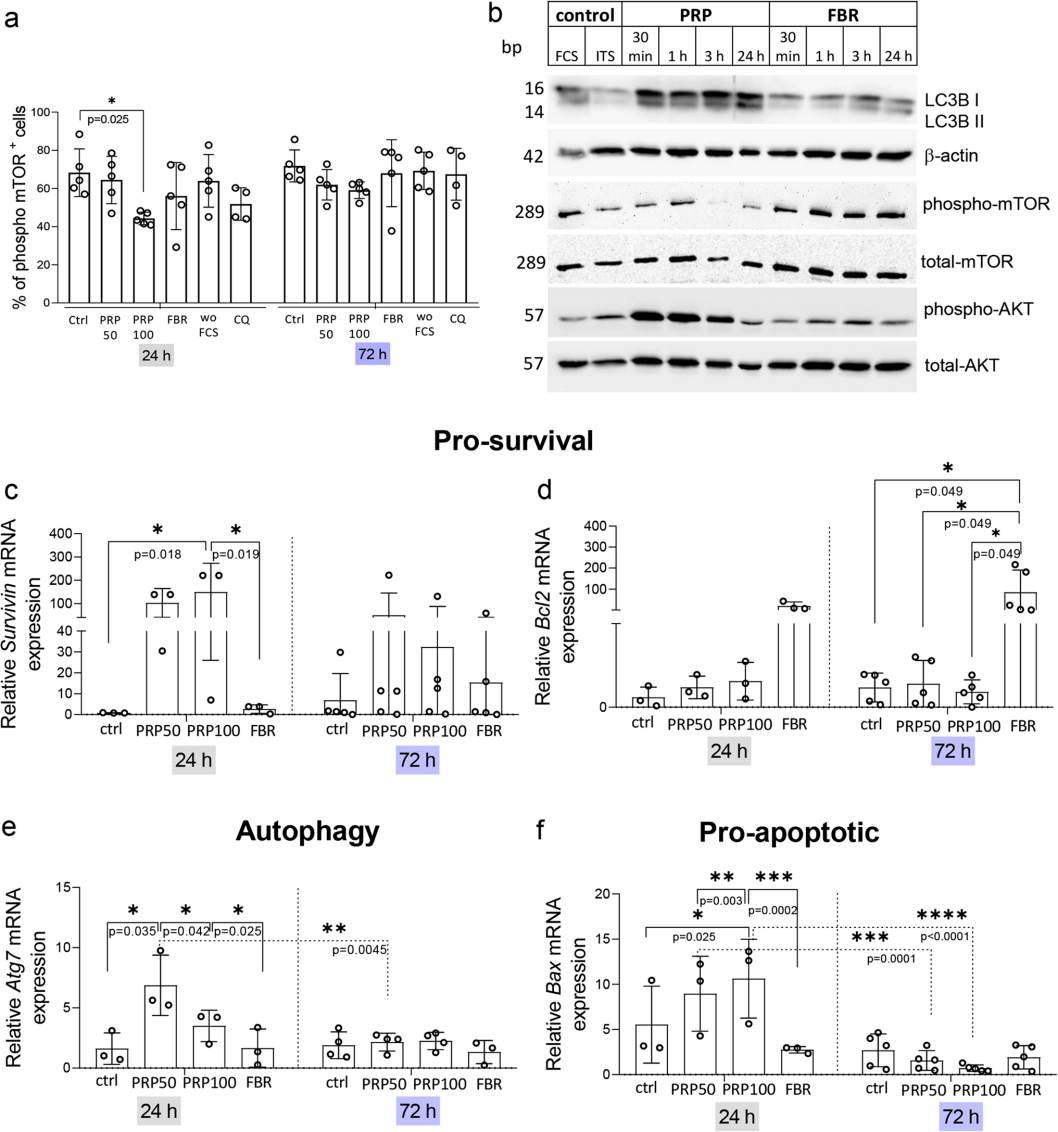
PRP stimuli modulate AKT and mTOR phosphorylation and regulate pro-apoptotic and autophagy-related genes. **a**) Flow cytometry analyses of phospho-mTOR in MSCs recovered after 24 h or 72 h of culture in hydrogels. (**b**) Representative immunoblots of time-dependent effects of PRP100- and FBR-hydrogel-derived CM on MSCs. LC3B I and II, phospho- and total-AKT, and phospho- and total-mTOR were analyzed at different time points. β-actin served as protein loading control. Gene expression of **c**) *Survivin*, **d**) *Bcl2*, **e**) *Atg7* and **f**) *Bax* determined at 24 h and 72 h upon culture of MSCs in standard conditions or hydrogels. Relative values to MSC culture in SGM without FCS were calculated for each group. Results are presented as mean ± SD. For comparison between groups, Kruskal–Wallis and two-way ANOVA with Bonferroni's multiple comparisons test was used to compare to the control and within groups in respect to the unexposed control sample, where *p < 0.05, **p < 0.01, ***p < 0.001 and ****p < 0.0001 indicate significant differences

mTOR is phosphorylated at Ser2448 via the PI3 kinase/AKT signaling pathway and phospho-mTOR inhibits the autophagic process [31]. Analyses of mTOR phosphorylation, showed a reduced percentage of MSCs with phospho-mTOR when retrieved from PRP-hydrogels, which was to a certain extent also observed in presence of CQ (as a potent inhibitor of mTOR signaling) after 24 h of treatment/incubation (Fig. 4a). This result was confirmed by Western blot analyses after treating MSCs with CM from PRP-hydrogels for 24 h, where we observed a decrease in phospho-mTOR (Fig. 4b). In addition, we found that PRP100-hydrogel derived CM induced the expression of LC3B (isoforms I and II) protein. We further observed increased phosphorylation of AKT 30 min after the treatment, while it returned to baseline levels after 24 h. These time-dependent changes in protein expression were not observed in MSCs treated with FBR-hydrogel-derived medium (Fig. 4b). These results indicate that PRP-derived stimuli might regulate AKT and mTOR signaling pathways with potential repercussions on the survival and activation of MSCs. In line with this, we found significantly increased gene expression for molecules involved in regulation of cell survival, autophagy and cell death in PRP-MSCs. In FBR-MSCs anti-apoptotic *Bcl2* gene expression was increased after 72 h (Fig. 4d). However, the expression of cell survival and death molecules by PRP-MSCs were more complex. PRP-MSCs expressed increased levels of the pro-survival gene *Survivin*, the autophagy-related gene *Atg7* and the pro-apoptotic *Bax* after 24 h. Interestingly, upregulated mRNA expression of *Atg7* and particularly *Bax* after 24 h were significantly abolished or reduced after 72 h, respectively, while this was not the case for *Survivin* (Fig. 4c–f). These results suggested time-dependent effects of PRP-hydrogels, where MSC survival was transiently challenged at the transcriptional level. Although these effects can be overcome in terms of total cell survival if MSCs continue to be cultured alone (SFig. 1b–d), they might participate in senescence induction and “apoptotic priming” of PRP-MSCs that might contribute to their death in communication with PBMCs.

### Clonogenic Growth and Metabolic Features of PRP-MSCs are Challenged

**Fig. 5:**
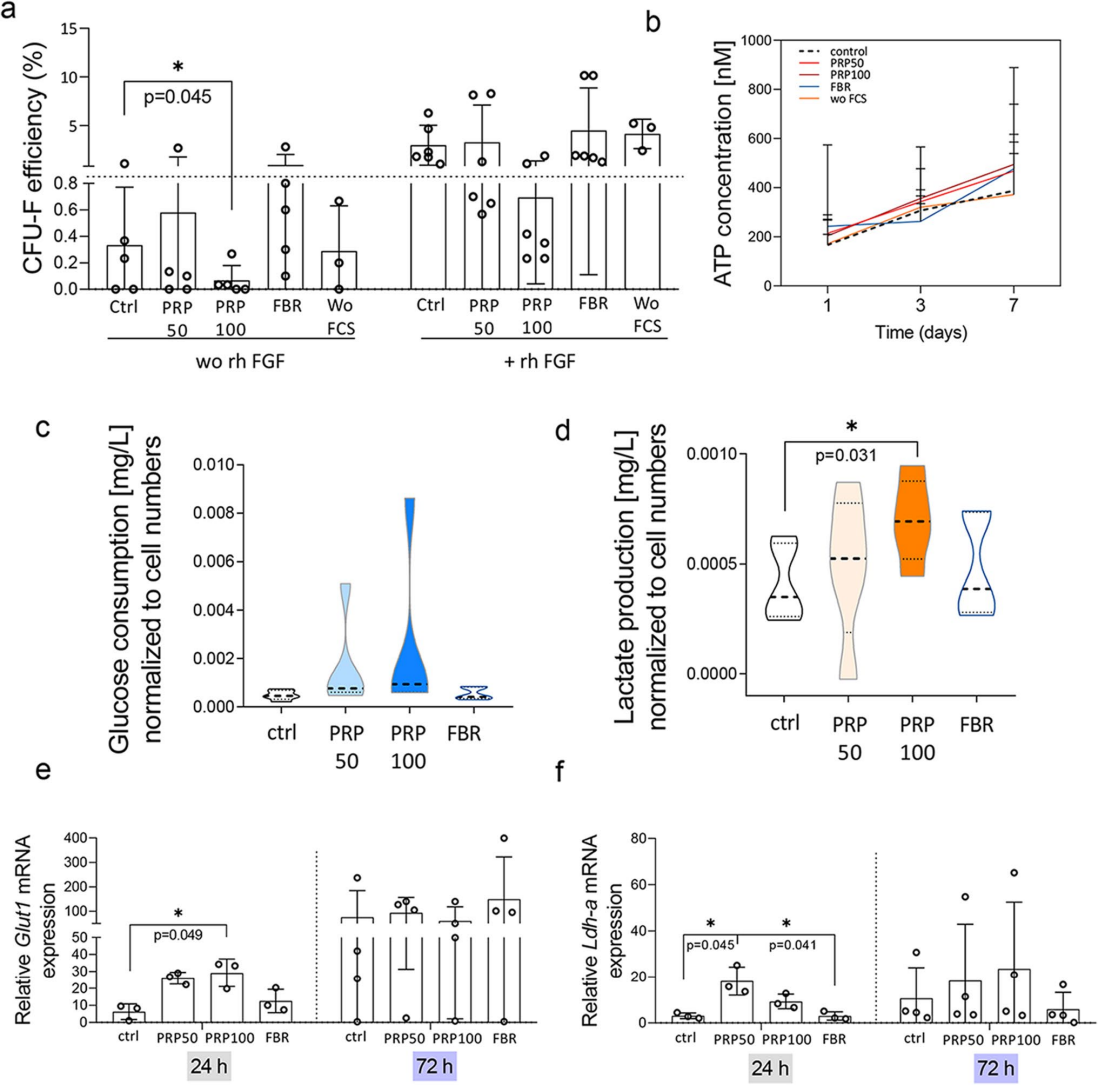
Cell growth and metabolism of MSCs recovered from PRP and FBR-hydrogels. **a**) Clonogenic activity of MSCs recovered from hydrogels. **b**) ATP levels in MSCs recovered from hydrogels and subsequently cultured for 1, 3 and 7 days. **c**) Glucose consumption and **d**) lactate production per cell determined at the end of their 72-h cultivation in hydrogels. Gene expression of **e**) *Glut-1* and **f**) *Ldh-a* estimated at 24 h (*n* = 3) and 72 h (*n* = 4) upon culture of MSCs in standard conditions or hydrogels. Relative values to MSC culture in SGM without FCS were calculated for each group. Results are presented as mean ± SD. For comparison between groups, Kruskal–Wallis and two-way ANOVA with Bonferroni's multiple comparisons test was used to compare to the control and within groups in respect to the unexposed control sample, where *p < 0.05 indicates significant differences

**Fig. 6:**
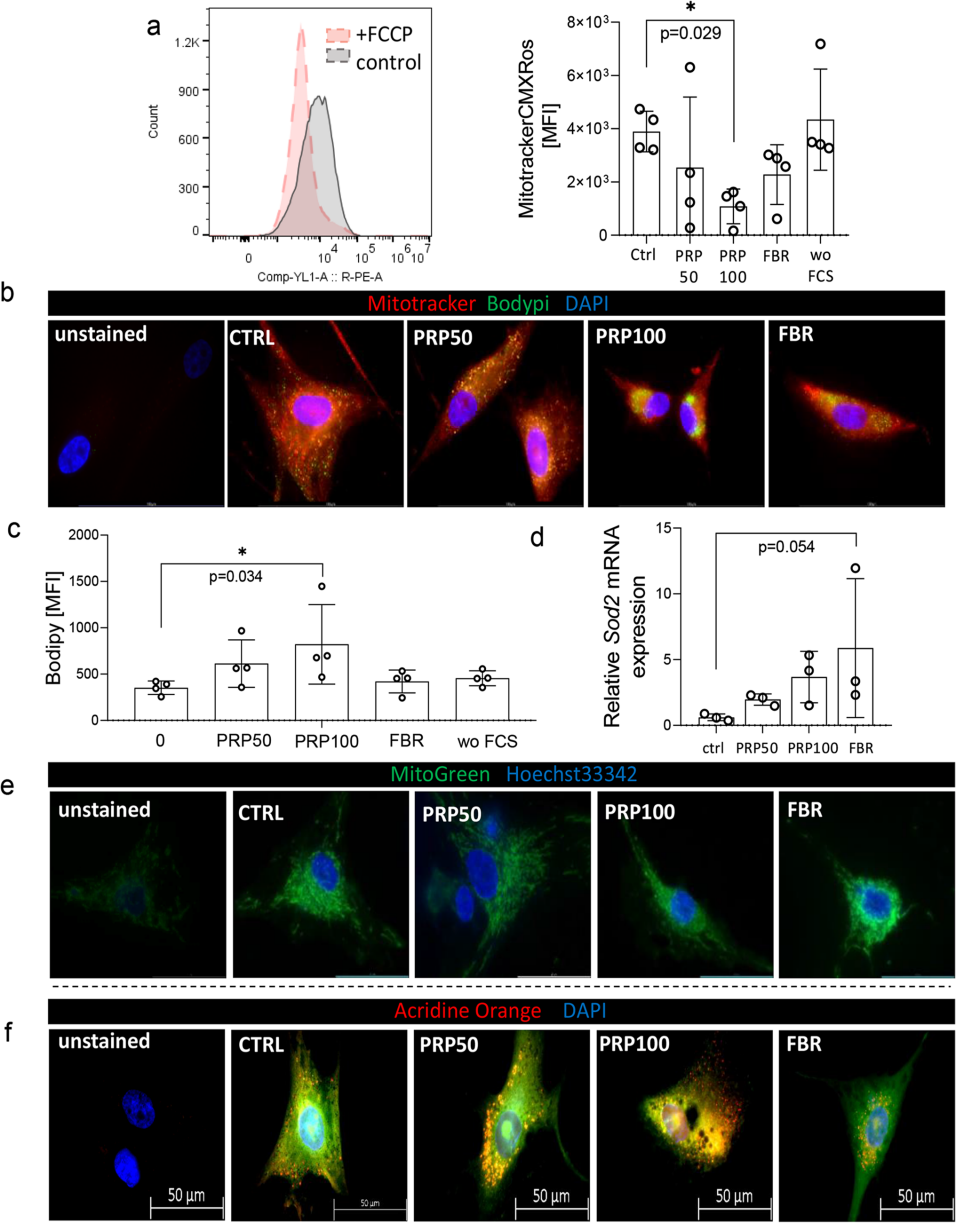
Mitochondrial profile and acidic vesicle formation in MSCs. **a**) Mitochondrial membrane potential estimated in MSCs upon their cultivation in hydrogels. Representative images of **b**) mitochondrial membrane potential-sensitive staining (MitoTrackerCMXRos) and neutral lipids (Bodipy) in recovered and fixed MSCs (*n* = 4). **c**) Neutral lipid distribution within MSCs. **d**) Gene expression of *Sod2* measured at 72 h (*n* = 3) upon culture of MSCs in standard conditions or hydrogels. Relative values to MSC culture in SGM without FCS were calculated for each group. **e**) Representative images of mitochondrial mass-sensitive staining and **f**) acridine orange fluorescence in MSCs recovered from gels where red signals indicate acidified regions (*n* = 3). Results are presented as mean ± SD. For comparison between groups, Kruskal–Wallis and two-way ANOVA with Bonferroni's multiple comparisons test was used to compare to the control and within groups in respect to the unexposed control sample, where *p < 0.05 indicates significant differences

The presence of stem cell-like entities is relevant for the self-renewal and maintenance of the entire bulk MSC population, also determining their subsequent capacity for long-term proliferation and osteogenic, chondrogenic or adipogenic differentiation. We assumed that clonal growth of recovered MSCs might be impacted by their previous exposure to hydrogels, and indeed the clonogenic potential of recovered PRP100-MSCs was reduced in absence or presence of rhFGF, which implies their impaired long-term maintenance. Significantly, PRP100-MSCs showed lower clonogenic activity in absence of rhFGF, when compared to all other tested groups. This was not observed in FBR-MSCs (Fig. 5a). In addition, we tested the osteogenic, adipogenic and chondrogenic differentiation potential of MSCs. With exception of a slight increase in ALP activity in FBR-MSCs upon osteogenic differentiation, we did not observe any significant effect of the hydrogels (SFig. 3a–d). Although ATP content of MSCs measured after their cultivation in hydrogels appeared to be unchanged (Fig. 5b), analyses of CM obtained at the end of MSC culture in hydrogels, showed that the consumption of glucose was slightly increased in PRP-MSCs (Fig. 5c). On the other side, we found a significant elevation of lactate production in cultures of PRP100-MSCs (Fig. 5d), suggesting a certain acidification and possibly, anaerobic nature of the environment, which might occur upon stress induction or starvation. In accordance with this, our results showed increased *Glut-1* and *Ldh-a* mRNA expression by PRP-MSCs when compared to control or FBR-MSCs (Fig. 5e, f). In addition, we investigated mitochondria functionality, and found that the culture of MSCs in PRP-hydrogels may provoke a significant decrease of the mitochondrial membrane potential (ψ_m_) of these cells, indicating a disturbed respiration of cells (Fig. 6a, b). Nevertheless, we did not observe any remarkable effect of hydrogels on the mitochondrial mass in MSCs (Fig. 6e). Beside this, we observed higher neutral lipid droplet accumulation in MSCs cultured in PRP-hydrogels when compared to control and the other groups (Fig. 6b, c).

Since formation of LC3-II positive vacuoles might coincide with autophagosome interaction with lysosomes, we next explored the presence of acidic vacuoles in MSCs upon their recovery from hydrogels. We observed that MSCs cultured in PRP-hydrogels contained more acidic vacuoles than MSCs in control and FBR groups (Fig. 6f). Yet, FBR-MSCs showed increased mRNA levels of mitochondrial superoxide dismutase (SOD)-2 (Fig. 6d), which suggests a stimulated anti-oxidative activity in these cells. Taken together, these results propose that although the in vitro short-term proliferation and differentiation potential of MSCs was not affected by the hematoma mimicking PRP-hydrogel, these cells might be exposed to certain metabolic stress. This may support the survival of specific subsets of non-clonogenic cells within the heterogeneous MSC population that might have limited regenerative potential. These findings suggest that the hematoma microenvironment might have a stressing nature that in turn affects stem cell populations during early bone healing phase.

## Discussion

In this study, we applied hematoma-mimicking PRP-hydrogels, as reliable models for the inflammatory phase of the healing process of tissues such as bone, in comparison to FBR-hydrogels without the hematoma-specific release of factors from platelets. With this, we aimed to evaluate the impact of such an environment on the immunomodulatory potential of MSCs that is not yet well explored in the context of bone healing. Results indicated that the PRP-hydrogels stimulated immunosuppressive functions of MSCs, which was associated with an increased susceptibility to cell death in communication with PBMCs. We found that the PRP-hydrogel environment impaired clonal growth and induced a senescent profile of PRP-MSCs. Although without impacting EV production by MSCs, PRP-hydrogels might represent a metabolically stressful environment, inducing acidification of MSCs and reducing the polarization of the mitochondrial membrane. In addition, stimuli derived from PRP-hydrogels lead to a reduced mTOR phosphorylation in MSCs, which might indicate involvement of AKT and mTOR signaling in observed changes in MSC immunosuppressive activity, growth, survival and metabolism. Eventually, the impact of PRP-hydrogels on apoptosis, senescence, metabolism and clonogenic growth of MSCs appears to be PRP concentration dependent, where PRP50 appears to be the better option for priming of MSCs. Thus, for targeted support of MSC immunosuppressive functions without affecting cell homeostasis, the PRP concentration should be optimized. This is of paramount importance for basic research of pro-regenerative effects of PRP and MSCs as well as their application in translational studies.

Augmentation of immunoregulatory functions of MSCs by PRP-hydrogels was accompanied by their increased apoptosis upon co-culture with PBMCs. Autophagy and apoptosis, both as part of the cell death program, can be involved in the regulation of MSC functions through the interplay of autophagy-related proteins (ATGs) and caspases [25]. The fact that MSCs upon in vivo administration often undergo fragmentation and cell death is well known [32], but it seems that apoptotic MSCs undergo efferocytosis and polarize monocytes into an immunosuppressive phenotype [33]. Moreover, cell death program appears to be an important regulatory point of immunomodulatory functions of MSCs, and it has been proposed that apoptosis is required for MSC-mediated immunoregulation, where deletion of the apoptotic effectors, BAK and BAX molecules, reduced MSC immunosuppressive effects in disease models. In this case, apoptosis of MSCs and their subsequent efferocytosis induced changes in metabolic and inflammatory pathways in alveolar macrophages that in turn affected immunosuppression and reduced disease severity [34]. Thus, it is reasonable to re-consider the immunosuppressive activity of MSCs as their context-dependent and provoked rather than intrinsic feature. On the contrary, we did not observe significant changes in the differentiation potential of MSCs after their cultivation in 3D hydrogels. Thus, distinct strategies for the improvement of lineage specification or immunosuppressive function of MSCs should be considered for their further application in clinical studies. Particularly, this is very important for understanding of realistic long-term capacity of MSCs to completely restore functions and structure of damaged tissue [35]. In addition, obtained results also support the previously recognized issues of determining effective MSC cell sources, times and routes of administration as well as minimal effective dosages for the treatment of damaged tissue [36]. Therefore, our results might also contribute to a better prediction of possible long-term adverse reactions to PRP and MSC administration.

The culture of MSCs with PRP has been reported to increase MSC proliferation and even differentiation potential in many studies [5, 16, 37, 38]. Moreover, it has been reported that embedding of rat bone marrow MSCs in PRP-hydrogels had a positive impact on their proliferation potential and ALP activity in 3D conditions [19]. We did not observe similar effects in our study. A possible explanation for this can be the specific nature of investigated cells in terms of their tissue-origin, patient (donor) state as well as the thrombocyte content and preparation protocol of PRP. It is also possible that in our setting 3D-imposed changes disappeared upon MSC recovery and subsequent monolayer culture. Also, in our study we exposed MSCs to the PRP- or FBR-hydrogel environment for 72 h, and so, we cannot exclude the possibility that a set of changes at the molecular and functional level might occur at earlier time points or afterwards.

Details regarding the influence of PRP and related products on immunomodulatory properties of MSCs are still scarce and inconsistent. There are evidences suggesting that PRP-derived factors provoke expression and secretion of pro-inflammatory proteins by MSCs [16], without observed anti-inflammatory activity [39]. On the other side, it was shown that PRP could reduce the secretion of interleukin-1β, interleukin-6 and tumor necrosis factor-α by MSCs [3, 38]. Our recent study also confirmed that PRP conditioned medium containing high amounts of the chemokine CXCL7 and pro-inflammatory RANTES, increased the immunosuppressive activity of MSCs [18]. This might explain the here observed upregulated immunosuppressive features of PRP-MSCs. Our results further showed that PRP-derived stimuli can upregulate AKT phosphorylation in MSCs and this is in accordance with a previous report [40]. Eventually, this might contribute to the regulation of survival-related genes in PRP-MSC as previously suggested [41]. Activation of downstream targets of phospho-AKT can stimulate a proliferative response of cells [41]. However, further research will have to elucidate whether AKT phosphorylation in our system appeared only as a transient event and/or as a compensatory response to the survival challenge provoked by PRP stimuli. Also, for the first time, the here presented results showed the potential of a PRP-hydrogel to reduce mTOR phosphorylation at Ser2448 in MSCs, which is known to be a mediator of cellular proliferation, metabolism, and survival [42]. In contrast, another study indicated that PRP elevated the levels of both phospho- and total-mTOR in keratinocytes [43], but without providing details regarding dynamic changes of phosphorylation levels. Since mTOR is the master regulator of autophagy, we assumed that this process might be involved in PRP-mediated effects in MSCs. This was confirmed by an increased expression of LC3-II protein after 72 h and *Atg7* mRNA after 24 h of PRP-hydrogel cultivation. Other studies suggested that PRP might promote cell migration, proliferation, and osteogenic differentiation, by activating autophagy in dental stem cells [44, 45]. Likewise, it has been found that PRP increased autophagy in osteoarthritic chondrocytes which in turn protected them from apoptosis [46]. Also, platelet lysate promoted autophagy via the AMPK/mTOR pathway in MSCs and in vivo data showed that administration of platelet lysate and MSCs simultaneously, exerted beneficial effect in osteoarthritic rat joints [47]. Beside an influence on autophagy, we here observed an PRP-increased expression of the pro-apoptotic *Bax* gene after 24 h, along with an increased caspase 3 and 7 activity. This indicates that at that time point the apoptotic events are efficient enough to support MSC death in communication with PBMCs. It is also possible that the here observed mitochondrial stress in PRP-MSCs induced caspase 3 and 7 activity, as described before [48, 49]. Furthermore, aforementioned caspases can interplay with autophagy proteins and thus be involved in autophagy regulation [25, 50]. Namely, mitochondria have a crucial role in the control of apoptosis, where a loss of mitochondrial membrane potential contributes to cell death by disruption of normal mitochondrial function and the release of apoptotic proteins BAX and BAK [49, 51]. However, further investigations are necessary to reveal whether and how cellular localization of these molecules participates in the here observed effects of PRP-hydrogel on apoptosis regulation in MSCs.

In our study, we did not observe a significant impact of the FBR-hydrogel on immunomodulatory properties of MSCs. Instead, FBR-hydrogel augmented the clonal growth of MSCs and maintained their metabolism and survival rate during communication with PBMCs. However, FBR-hydrogel reduced the production of CD63^+^ and CD81^+^ EVs by MSCs. Both EV biogenesis and release are multistep processes [52] where the latter one includes the participation of LC3-IIprotein [53]. Therefore, additional investigations would be necessary to clarify the mechanism of FBR-prevented EV production in MSCs and its’ repercussions on cell homeostasis and clonal growth of MSCs. In addition to this, FBR-MSCs displayed elevated levels of anti-apoptotic B-cell lymphoma-2 (*Bcl-2*) and mitochondrial anti-oxidative enzyme *Sod-2 *expression. Thus, it is possible that FBR-hydrogels might contribute to the maintenance of fundamental stem cell features devoid of “apoptotic priming” insults and associated immunomodulatory activity observed in PRP-MSCs. Previous studies suggested positive effects of fibrinogen-based coating systems in terms of MSC viability and proliferation [54], but also maintainance of stem cell markers in induced pluripotent stem cells, such as SSEA4, Oct3/4, TRA1-60 and NANOG [55]. The here observed preservation of clonal growth of FBR-MSCs is in line with our previous report where BM mononuclear cell fractions cultured in fibrin gels for up to 14 days, showed a higher clonogenic potential after recovery from gels than cells seeded without fibrin embedding [56].

Another important aspect identified in our study is the metabolic perturbation in MSCs exposed to the PRP-hydrogels that might cause the here observed cellular functions. It is known that the mTOR pathway impacts nutrient uptake and anabolic processes [31], where in our study we found reduced mTOR phosphorylation levels in PRP-MSCs. The here detected increased glucose uptake and lactate production along with increased *Glut-1* and *Ldh-a* gene expression in PRP-MSCs are in line with previous findings where the active mTOR pathway regulated glucose utilization and M2 polarization of macrophages [57]. Also, recent studies revealed that PRP-stimulated survival of damaged MSCs occurred due to alterations in the energetic (oxidative) metabolism [6, 58]. However, observed changes in PRP-MSCs can be also related to the observed impaired mitochondrial ψ_m_ and possible cell respiration. Acidification of the environment and MSCs itself suggest potential nutrient stress and oxygen deprivation observed in cells when cultured in PRP-hydrogels, which again, was not the case in FBR-hydrogels. In parallel with the upregulation of *Glut1,* acidification in hematoma has been likewise shown in vivo in rat and sheep models [59], which is in accordance with our findings.

To find the best in vitro model to mimic in vivo fracture conditions, a previous study applied combined restriction of oxygen and nutrient supply (representative for the bone marrow microenvironment per se) to support survival of immune cells [60]. Moreover, along with hypoxia-induced and angiogenic markers, acidification-related *Ldh-a* gene was also found to be increased in an oxygen-restricted in vitro hematoma model [61]. With regards to these findings, we cannot exclude that the here observed PRP-hydrogel effects on MSCs are at certain extent consequence of the reduced oxygen or nutrient delivery in the 3D environment. However, we did not observe similar changes in FBR-MSCs implying that the activity of platelet-released factors had a more dominate effect than 3D hydrogel-related limitations in supply with oxygen or nutrients. In parallel with acidification of the environment, the increased accumulation of lipid droplets in PRP-MSCs shown in our study, based on previous studies appears to be rather involved in gaining of an altered metabolic and immunoregulatory features [62] and senescent profile [61], than in adipogenic differentiation. Importantly, adaptation of the energetic metabolism has been recognized as a feature of MSCs involved in regulation of immunomodulatory activities. A shift to aerobic glycolysis [63] induced by hypoxia, inflammatory compounds or mTOR pathway modulators [64] has been shown to contribute to immunoregulatory features of MSCs [65]. Therefore, we can assume that the metabolic re-configuration observed here contributes to the immunomodulatory effects achieved by PRP-MSCs.

A previous study reported a rejuvenating potential of platelet lysate, where a reduction of β-galactosidase in senescent MSCs was observed [66]. This is in contrast with our findings of increased β-galactosidase activity in MSCs cultured in PRP-hydrogels. Although a recent study demonstrated senescence-associated upregulation of EV biogenesis along with autophagy [67], this recycling pathway may also participate in the clearance of EVs [44]. We can assume that PRP-hydrogels induced a senescence profile in PRP-MSCs, simultaneously leading to autophagy program, which was not observed in FBR-MSCs. In aged MSCs autophagy is involved in the regulation of their impaired differentiation and bone loss [68]. Thus, it can be speculated that reduced mTOR phosphorylation and the PRP-stimulated senescent phenotype of MSCs shown in our study can contribute to their immunomodulatory functions, rather than to affect differentiation. In general, senescence-associated EV production is considered to protect cells from excessive inflammatory responses [69]. In accordance with this, we here showed a reduced β-galactosidase activity in FBR, along with a reduced release of CD63^+^ and CD81^+^ EVs by MSCs, suggesting that cellular homeostasis was more protected during MSC culture in FBR-hydrogels. Finally, we cannot exclude the possibility that PRP-promoted senescence and the associated secretome of MSCs is of importance for the achieved immunomodulatory effects.

## Conclusions

This study brings novel evidence on the impact of hematoma components on immune and regenerative features of bone marrow MSCs, providing insights into not yet highlighted metabolic and molecular targets for the future management of the tissue healing process. Obtained results indicate that although PRP stimulated immunosuppressive functions of MSCs, it also upregulated their susceptibility to cell death. Moreover, PRP impaired clonal growth of MSCs and induced a senescent profile simultaneously with reduced mTOR phosphorylation and induced acidification of MSCs. Thus, the coincidence of cell stress, apoptosis and pro-survival mechanisms suggests a complex network of events mediated by PRP-derived factors. For the first time, we showed that pleiotropic PRP-related stimuli might represent a survival challenge and “apoptotic priming”, that are detrimental for stem cell-like growth of MSCs and important for their therapeutic consideration.

## Supplementary Information

Below is the link to the electronic supplementary material.

Supplementary file1 (DOCX 1352 KB)

## Data Availability

All data generated during this study are included in this published article and its supplementary information files.

## Declarations

### Study Approval

The study was performed according to the Declaration of Helsinki and was approved by the local ethical authorities. Human bone marrow samples were collected at Orthopedic Clinic “Koenig Ludwig Haus” at the Department of Orthopedic Surgery, Wuerzburg under a protocol approved by Ethical Committee with (187/18). All participants signed informed consent.

## Abbreviations

BM: bone marrow
MSC: mesenchymal stromal cell
PRP: platelet-rich plasma
FBR: fibrin
EV: extracellular vesicle
SDF: stromal-derived factor
PDGFs: platelet-derived growth factors
PL: platelet lysate
DAMPs: damage-associated molecular patterns
TLRs: Toll-like receptors
CM: conditioned medium
PRP-MSCs: MSCs cultured in PRP-hydrogel
FBR-MSCs: MSCs cultured in FBR-hydrogels
PBS: phosphate-buffered saline
FCS: fetal calf serum
CFU-F: colony forming unit-fibroblastic
P: passage
SGM: standard growth medium
rh: recombinant human
STS: staurosporin
CQ: chloroquine diphosphate
PPP: platelet-poor plasma
NaCl: sodium chloride
CaCl_2_: calcium chloride
PBMCs: peripheral blood mononuclear cells
LGM: lymphocyte growth medium
CFSE: carboxyfluorescein-diacetate *N*-succinimidyl-ester
PHA-L: phytohemagglutinin-L
IFN: interferon
PMA: phorbol 12-myristate 13-acetate
PE-Cy7: phycoerythrin-Cy7
PE: phycoerythrin
APC: allophycocyanin
FITC: fluorescein
NaN_3_: sodium azide
LC3b: microtubule-associated protein 1A/1B light chain 3B
COX2: cyclooxygenase-2
DAPI: 4′,6-diamidino-2-phenylindole dihydrochloride
AO: acridine orange
FCCP: carbonyl cyanide 4-(trifluoromethoxy)phenylhydrazone
MTG: MitoTracker Green fluorescent probe
ATP: adenosine triphosphate
β-gal: β-galactosidase
*B2m*: β2-microglobulin
*Ptgs2*: prostaglandin-endoperoxide synthase 2
(*Il*)-6: interleukin 6
FVD: fixable viability dye
CD: cluster of differentiation
*p16*: cyclin-dependent kinase inhibitor 2A
mTOR: mammalian target of rapamycin
*Atg7*: autophagy related protein 7
-*Bcl2*: B-cell lymphoma 2
*Bax*: BCL2 associated X
*Glut-1*: glucose transporter 1
*Ldh-a*: lactate dehydrogenase A
ψm: mitochondrial membrane potential
Sod-2: superoxide dismutase-2
