# Supplementary material for: Enhancement of Immunosuppressive Activity of Mesenchymal Stromal Cells by Platelet-Derived Factors is Accompanied by Apoptotic Priming
Source: Stem Cell Rev Rep. 2022 Nov 22;19(3):713–33. doi: 10.1007/s12015-022-10471-4 (PMC10070232; doi:10.1007/s12015-022-10471-4)
Supplement: Supplementary file 1 — Supplementary file1 (DOCX 1352 KB) [file 12015_2022_10471_MOESM1_ESM.docx]

**Supplementary Material**

**Enhancement of immunosuppressive activity of mesenchymal stromal cells by platelet-derived factors is accompanied by apoptotic priming**

Drenka Trivanovic ^1,2^, Noah Volkmann ^1,2^, Magdalena Stoeckl ^1,2^ Tobias Tertel ^3^, Maximilian Rudert ^4^, Bernd Giebel ^3^, Marietta Herrmann^1,2*^

^1^ IZKF Research Group Tissue Regeneration in Musculoskeletal Diseases, University Clinics Würzburg, Germany

^2^ Orthopaedic Center for Musculoskeletal Research, University of Würzburg, Germany

^3^ Institute for Transfusion Medicine, University Hospital Essen, University of Duisburg-Essen, Germany

^4^ Chair and Director, Orthopaedic Department, University of Würzburg, Germany

**Material and Methods:**

**Evaluation of MSC viability in cell growth media**

**Viability of MSCs.** MSCs were seeded at a concentration of 5 x 10^3^ cells/well in 96-well plates and cultured in SGM or LGM w/o PHA-L in standard conditions. After 72 h, 3-(4,5-dimethylthiazol-2-yl)-2,5-diphenyltetrazolium bromide (MTT, Sigma Aldrich, USA) was added at a final concentration of 50 µg/mL and cells were additionally incubated for 3 h. Formazan crystals were dissolved in DMSO and absorbance was read at 540 nm at Tecan reader.

**MSC proliferation.** MSCs were seeded at a concentration of 1 x 10^5^ cells/well in 6-well plates and cell counting was performed after 72 h. Cells were cultured in SGM or LGM w/o PHA-L. MSCs were observed by Microscopy (Leica) and the number of recovered cells was determined by Trypan-blue exclusion test.

***In vitro* differentiation of MSCs**

To test differentiation, after their recovery from PRP and FBR hydrogels, MSCs were re-seeded in 96-well plates at a concentration of 10^4^ cells/well in triplicates and incubate overnight at standard conditions in SGM. Next day, SGM was replaced with appropriate osteogenic or adipogenic differentiation medium as stated below.

**Osteogenesis**. SGM was replaced with low-glucose (D-glucose 1 g/L, Sigma Aldrich) osteogenic differentiation control medium (without chemical inducers) or containing differentiation cocktail (dexamethasone 10 nM, L-ascorbic acid-2-phosphate 50 µg/mL, β-glycerophosphate 5 mM (all from Sigma Aldrich)).

*Alkaline phosphatase activity assay*. MSCs cultured in osteogenic medium were assayed for ALP activity after 7 and 14 days. A colorimetric assay using Napthol as substrat of ALP which is dephosphorylated in acidic environment and Fast Blue RR (both from Sigma Aldrich) was used for visualization of ALP activity. Cells were washed with PBS, fixed in 4 % PFA at RT for 30 min, and then stained with 0.1 mol/L Tris Buffer pH 9.0 containing 0.6 mg/ml Fast Blue RR Salt and 0.2 mg/ml Naphthol AS-MX Phosphate Disodium Salt for 30 min at RT in the dark. Precipitates were dissolved in NaOH in 95% ethanol. Absorbance was read at 405 nm (Tecan reader, Tecan Trading AG, Switzerland).

*In vitro mineralization assay*. Osteogenic medium for 21-day cultured MSCs was discarded from each well and cells were fixed in ice-cold 70 % ethanol for 1 hour at -20 ˚C, followed by staining with Alizarin Red 2 % for 20 min. Deposits were disolved in 10 % cetylpyridinium cloride (Sigma Aldrich) and absorbance was read at 540 nm (Glomax, USA or Tecan reader, Tecan Trading AG).

**Adipogenesis**. SGM was replaced with high-glucose (D-glucose 4.5 g/L, Invitrogen) adipogenic differentiation control medium (without chemical inducers) or containing differentiation cocktail (dexamethasone 1 µM, 3-isobutyl-1-methylxanthine 500 µM, indomethacin 60 µM, rh insulin 5 µg/mL (all from Sigma Aldrich)). MSCs cultured in adipogenic medium were assayed for lipid droplet accumulation by using 0.3 % Oil Red O solution after 21 days. 100 % isopropanol was added to elute Oil Red O and after incubation for 10 min at shaker, absorbance was read at 405 nm at a Tecan reader (Tecan Trading AG).

**Chondrogenesis**. Recovered MSCs were seeded at a concentration of 2 x 10^5^ cells/tube in a 1.5 mL tube in SGM and centrifuged at 400 x *g* for 5 min. After 24 h, spheroid-like aggregates were treated with chondrogenic medium for 21 days. Chondrogenic control medium (CM) contained: high-glucose (D-glucose 4.5 g/L, Invitrogen) medium, 100 nM dexamethasone, 50 µg/mL L-ascorbic acid-2-phosphate, 100 µg/mL pyruvate, 40 µg/mL L-proline and 1 % insulin-transferrin-sodium selenite (all purchased from Sigma Aldrich). For chondrogenic differentiation medium, CM was additionally supplemented with fresh 10 ng/mL rhTGF-β (PromoCell, Germany). After 21 days, aggregates were fixed in 75% methanol, submerged in 5% sucrose for 24 h, embedded in O.C.T. (Sakura Finetek, CA), cut into sections (thickness 12 μm) and fozen at -20°C. Cryosections were stained with Fast Green (0.02%) and Safranin O (0.1%) for glycosaminoglycan (GAG) observation.

| Antibody / Probe | Company | | Catalogue Number | | Clone and Fluorochrome | | Source | | Reactivity | |
| --- | --- | --- | --- | --- | --- | --- | --- | --- | --- | --- |
| CD45 | eBioscience | | 47-0459-42 | | HI30 APC-efluor780 | | mouse | | human | |
| CD45 | eBioscience | | 69-0459-42 | | HI30 APC-efluor506 | | mouse | | human | |
| CD3 | eBioscience | | 46-0047-42 | | APC-efluor780 | | mouse | | Chimpanzee, Human | |
| CD3 | eBioscience | | 46-0047-42 | | SK7 FITC | | mouse | | Chimpanzee, Human | |
| CD4 | eBioscience | | 46-0047-42 | | SK3 PerCP-eFluor710 | | mouse | | human | |
| CD8a | eBioscience | | 25-0087-42 | | SK1 PE-Cyanine7 | | mouse | | human | |
| CD25 | eBioscience | | 17-0259-42 | | BC96 APC | | mouse | | human | |
| CD34 | eBioscience | | 11-0349-42 | | 4H11 FITC | | mouse | | human | |
| CD146 | eBioscience | | 12-1469-42 | | P1H12 PE | | mouse | | human, dog, mouse, rabbit | |
| CD73 | eBioscience | | 46-0739-42 | | AD2 PerCP-efluor710 | | mouse | | human | |
| CD13 | eBioscience | | 25-0138-42 | | WM-15 PE-Cyanine7 | | mouse | | human | |
| CD31 | eBioscience | | 25-0319-42 | | WM-59 PE-Cyanine7 | | mouse | | human | |
| HLA-DR | eBioscience | | 47-9956-42 | | LN3 APC-efluor780 | | mouse | | human | |
| CD80 | eBioscience | | 46-0809-42 | | 2D10.4 PerCP-efluor710 | | mouse | | human | |
| Phospho-mTOR (Ser2448) | eBioscience | | 25-9718-42 | | MRRBY PE-Cyanine7 | | mouse | | human | |
| IFN-gamma | eBioscience | | 48-7319-42 | | 4.SB3 efluor450 | | mouse | | human | |
| FOXP3 | eBioscience | | 12-4776-42 | | PCH101 PE | | mouse | | human | |
| Fixable Viability Dye | eBioscience | | 65-0865-14 | | efluor780 | | NA | | NA | |
| Fixable Viability Dye | eBioscience | | 65-0866-18 | | efluor506 | | NA | | NA | |
| LC3B | Cell Signaling Technology | | 2775 | | NA | | rabbit | | mouse, rat, human | |
| Lamin B1 | R&D Systems | | MAB8525 | | 919007 | | mouse | | human | |
| Cox-2 | Santa Cruz Biotechnology | | sc-166475 | | D-12 | | mouse | | mouse, rat, human | |
| Phospho-mTOR (Ser2448) | Cell Signaling Technology | | 5536 | | D9C2 | | rabbit | | mouse, rat, human | |
| mTOR | Cell Signaling Technology | | 2983 | | 7C10 | | rabbit | | mouse, rat, human | |
| Phospho-Akt (Ser473) | Cell Signaling Technology | | 4060 | | D9E | | rabbit | | mouse, rat, human | |
| Akt (pan-total) | Cell Signaling Technology | | 4691 | | C67E7 | | rabbit | | mouse, rat, human | |
| β-Actin | Cell Signaling Technology | | 3700 | | 8H10D10 | | mouse | | Human, Mouse, Rat, | |
| Goat Anti-Rabbit IgG H&L | Abcam | | ab150077 | | Alexa Fluor® 488 | | goat | | rabbit | |
| Goat Anti-Mouse IgG H&L | Abcam | | ab150116 | | Alexa Fluor® 594 | | goat | | mouse | |
| Goat Anti-Mouse IgG H&L | Abcam | | ab150113 | | Alexa Fluor® 488 | | goat | | mouse | |
| Anti-mouse IgG | Cell Signaling Technology | | 7076 | | HRP-linked | | horse | | mouse | |
| Anti-rabbit IgG | Cell Signaling Technology | | 7074 | | HRP-linked | | goat | | rabbit | |
| CFSE | Sigma Aldrich | | 21888 | | NA | | NA | | NA | |
| CellTracker™ Green CMFDA Dye | Invitrogen | | C2925 | | NA | | NA | | NA | |
| MitoTracker™ Red CMXRos | | Invitrogen | | M7512 | | NA | | NA | | NA |
| MitoTracker® Green FM | | Cell Signaling Technology | | 9074 | | NA | | NA | | NA |
| Acridine Orange | | Sigma Aldrich | | 318337 | | NA | | NA | | NA |
| BODIPY™ 493/503 | | Invitrogen | | D3922 | | NA | | NA | | NA |

**Supplementary Table 1.** List of antibodies and probes used in the study.

**Supplementary Table 2**. List of forward and reverse primers used in experiments.

| Transcript | Forward primer (5'-3') | Reverse primer (5'-3') | NBCI sequence | size [bp] |
| --- | --- | --- | --- | --- |
| *B2m* | GATGAGTATGCCTGCCGTGT | TGCGGCATCTTCAAACCTCC | NM_004048.4 | 116 |
| *Glut-1* | CTGCTCATCAACCGCAAC | CTTCTTCTCCCGCATCATCT | NM_006516.3 | 129 |
| *Survivin (BIRC5)* | GACGACCCCATAGAGGAAC | CCTTTGCAATTTTGTTCTTG | NM_001168.3 | 136 |
| *p16* | GACTGGCTGGCCACGG | ATCATGACCTGGATCGGCCT | NM_001195132.2 | 120 |
| *Il-6* | CAATGAGGAGACTTGCCTGGT | CACAGCTCTGGCTTGTTCCT | NM_000600.5 | 112 |
| *Ptgs2* | CAAATTGCTGGCAGGGTTGC | AGGGCTTCAGCATAAAGCGT | NM_000963.4 | 125 |
| *Ldha-A* | ACGTGCATTCCCGATTCCTT | AACAGCACCAACCCCAACAA | NM_005566.4 | 130 |
| *Sod2* | GCTCCCCGCGCTTTCTTA | GCTGGTGCCGCACACT | NM_001322816.1 | 112 |
| *Bcl-2* | GATTGTGGCCTTCTTTGAG | CAAACTGAGCAGAGTCTTC | NM_000633.2 | 121 |
| *Bax* | TCCCCCCGAGAGGTCTTTT | CGGCCCCAGTTGAAGTTG | NM_004324.4 | 109 |
| *Atg7* | ACCCAGAAGAAGCTGAACGA | CTCATTTGCTGCTTGTTCCA | NM_001349238.1 | 106 |

**Supplementary Table 3**. Expression of surface markers by MSCs cultured in PRP- and FBR-hydrogels for 72 h.

| **Antigen** | **Ctrl** | **PRP50** | **PRP100** | **FBR** |
| --- | --- | --- | --- | --- |
| CD13 | 87.65±15.38 | 94.15±4.07 | 90.12±7.93 | 84,07±19.74 |
| CD44 | 90.58±9.63 | 89.7±6.005 | 85.47±9.72 | 83.52±17.004 |
| CD90 | 77.7±15.6 | 72.37±17.83 | 65.78±14.08 | 68.35±19.77 |
| CD105 | 86.52±13.35 | 90.63±4.85 | 86.18±4.84 | 84.62±15.3 |
| CD73 | 81.13±19.05 | 84.78±5.04 | 80.4±4.447 | 77.12±23 |
| CD146 | 2.33±3.416 | 4.34±4.11 | 5.41±5.34 | 1.83±2.58 |
| CD31 | 2.54±2.32 | 2.65±0.82 | 7.57±6.25 | 9.60±10.74 |
| CD34 | 1.24±1.21 | 0.68±0.58 | 0.45±0.34 | 0.99±0.67 |
| CD80 | 2.44±1.89 | 1.65±1.16 | 1.21±0.68 | 3.14±0.88 |
| HLA-DR | 3.48±2.23 | 2.35±1.17 | 1.77±1.01 | 3.82±2.97 |


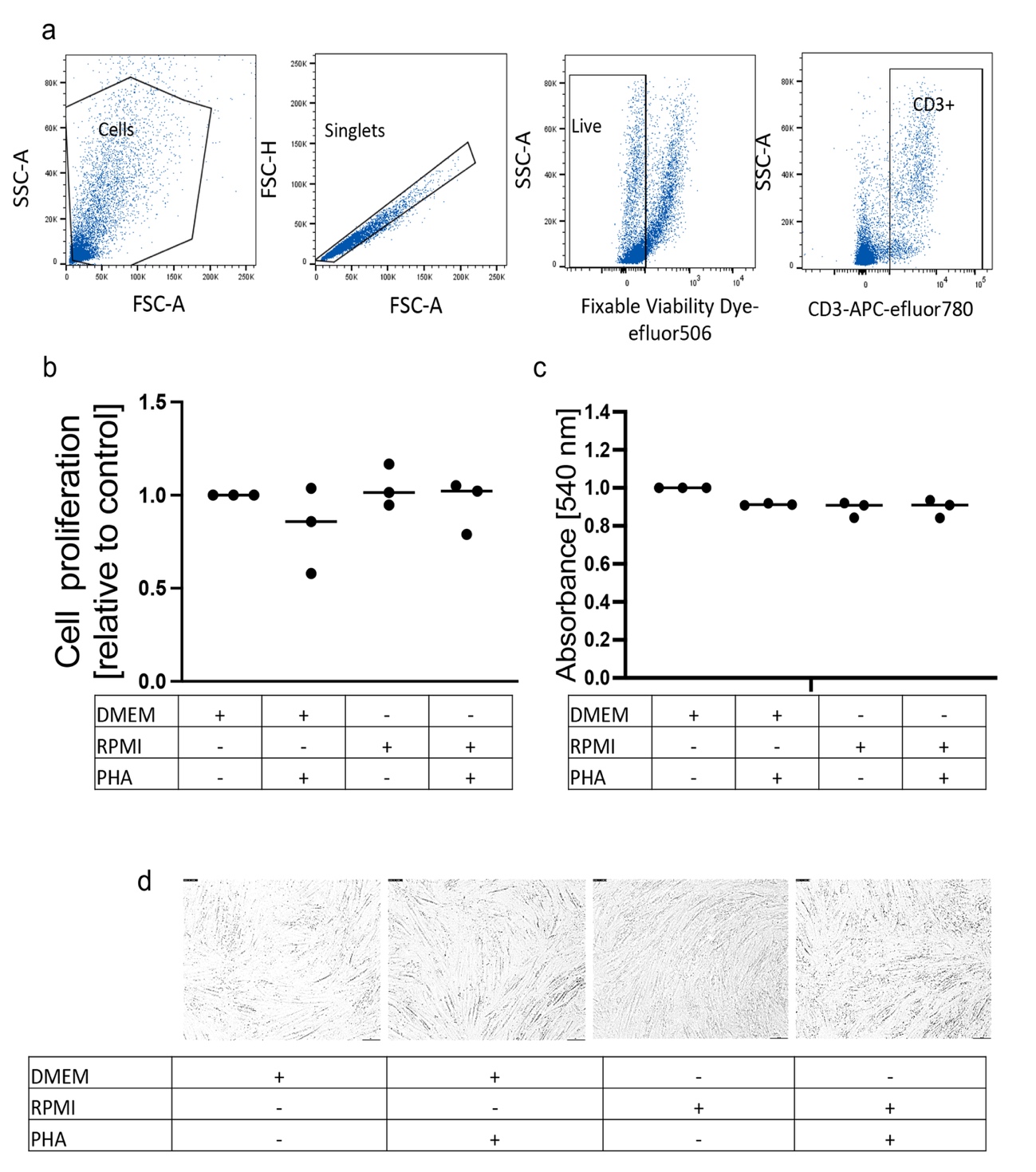


**Supplementary Figure 1.** a) Gating strategy applied for analyses T-lymphocyte phenotypes (steps before CD4 and CD8 analyses were shown). MSC b) number and c) viability and d) morphology estimated after 72 h cultivation in DMEM (SGM) or RPMI (LGM) w/o PHA-L (n=3). Results are presented as mean±SD. For comparison between groups, Kruskal-Wallis test was used to compare to control and within groups in respect to control sample (DMEM, SGM).c) Morphology of MSCs after 72 h of cultivation. Scale bars: 50 µm.

**
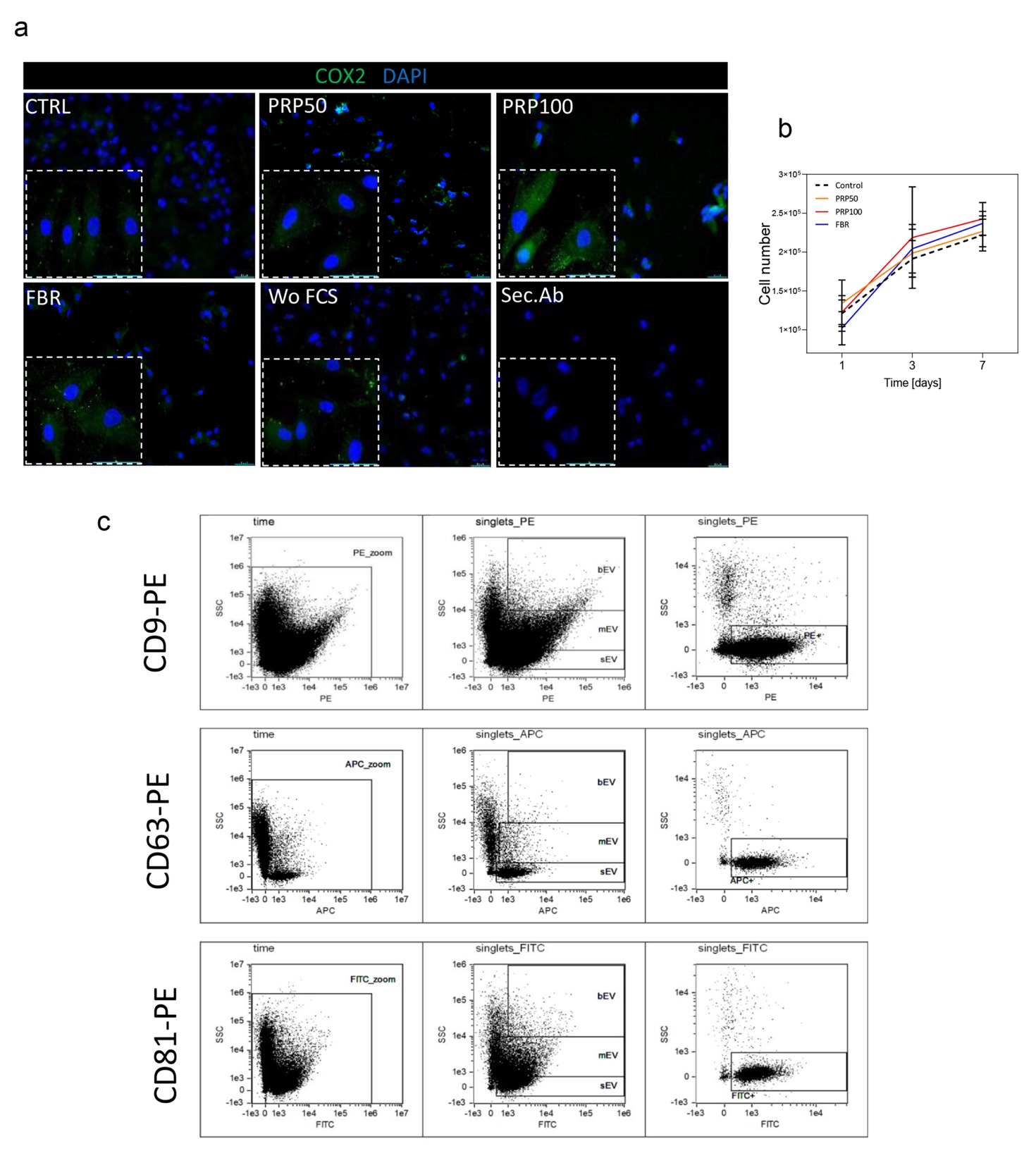
**

**Supplementary Figure 2.** a) Immunofluorescence images of COX-2 expression in MSCs recovered after 72-hours of culture in hydrogels. Representative photos of 3 independent experiments are shown. Scale bar = 50 μm. b) Proliferation of MSCs recovered from hydrogels and followed after 1, 3 and 7 days under standard conditions. Results are presented as mean±SD. For comparison between groups. Kruskal-Wallis was used to compare to control and within groups in respect to unexposed control sample. c) Gating strategy for imaging flow cytometry analyses of MSC-derived extracellular vesicles (big-bEV; medium-mEV; small-sEV) presented in obtained conditioned media.

**
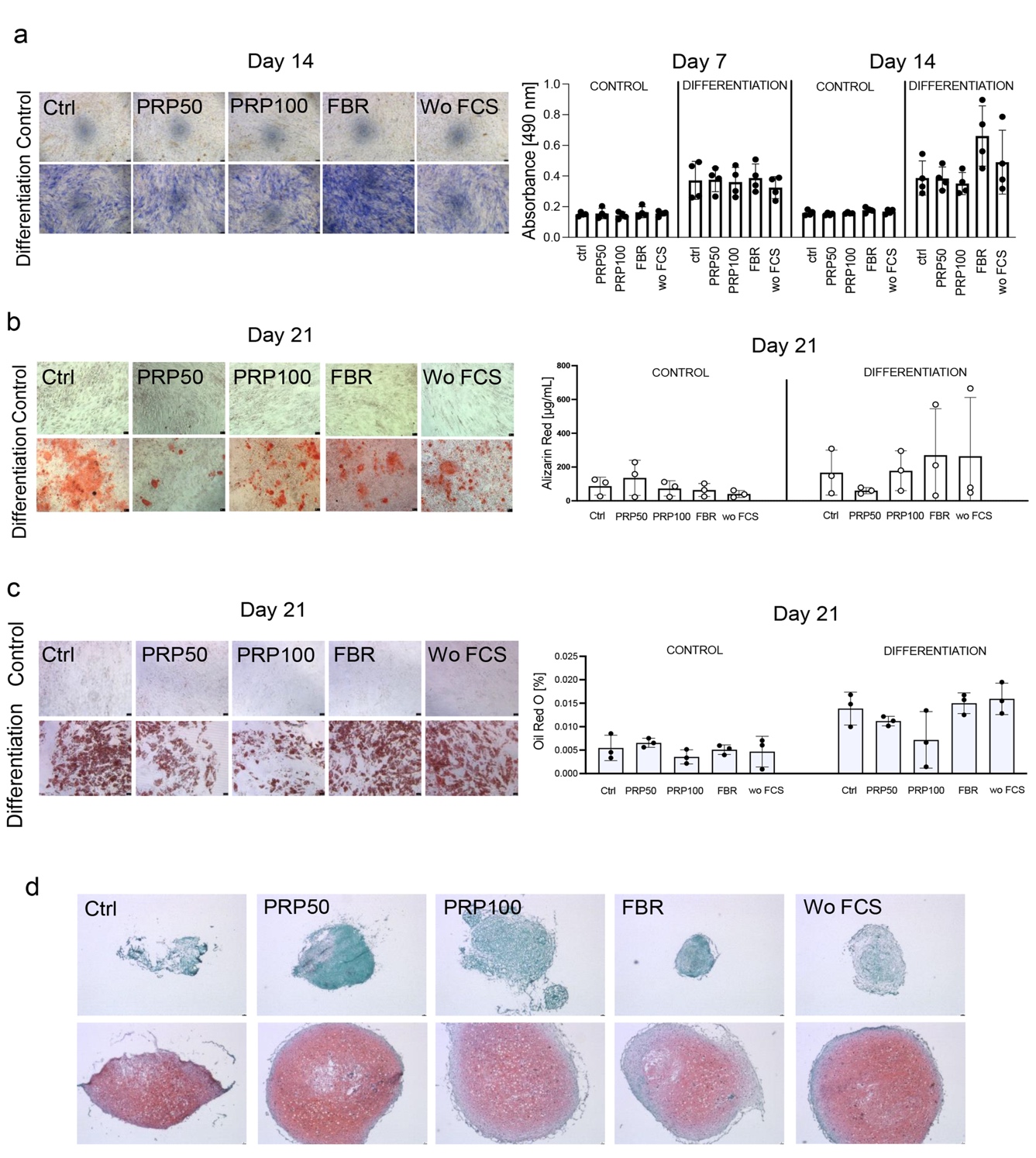
**

**Supplementary Figure 3**. **Differentiation potential of MSCs recovered from hydrogels**. a) Representative photos of ALP staining at day 14 are shown (n=4). b) Representative photos of Alizarin Red stainings are shown (n=3). c) Representative photos of Oil Red O staining are shown (n=3). d) Representative images glycosaminoglycan staining (Safranin-O) in MSC-formed spheroids (Scale bar = 100 µm) (n=2). Results are presented as mean±SD. For comparison between groups, two-way ANOVA with Bonferroni's multiple comparisons test was used to compare to control and within groups. Proliferation of MSCs recovered from hydrogels and followed after 1, 3 and 7 days under standard conditions. Results are presented as mean±SD. For comparison between groups. Kruskal-Wallis was used to compare to control and within groups in respect to unexposed control sample.
